# Comparative genomics and evolution of the amylase-binding proteins of oral streptococci

**DOI:** 10.1186/s12866-017-1005-7

**Published:** 2017-04-20

**Authors:** Elaine M. Haase, Yurong Kou, Amarpreet Sabharwal, Yu-Chieh Liao, Tianying Lan, Charlotte Lindqvist, Frank A. Scannapieco

**Affiliations:** 1 Department of Oral Biology, School of Dental Medicine, University at Buffalo, State University of New York, Buffalo, NY USA; 2 Division of Biostatistics and Bioinformatics, Institute of Population Health Sciences, National Health Research Institutes, Miaoli, Taiwan; 3 Department of Biological Sciences, University at Buffalo, State University of New York, Buffalo, NY USA; 4 Department of Oral Biology, School of Stomatology, China Medical University, Shenyang, People’s Republic of China

**Keywords:** Commensal, Adaptation, Amylase, Phylogenetics, Horizontal gene transfer

## Abstract

**Background:**

Successful commensal bacteria have evolved to maintain colonization in challenging environments. The oral viridans streptococci are pioneer colonizers of dental plaque biofilm. Some of these bacteria have adapted to life in the oral cavity by binding salivary α-amylase, which hydrolyzes dietary starch, thus providing a source of nutrition. Oral streptococcal species bind α-amylase by expressing a variety of amylase-binding proteins (ABPs). Here we determine the genotypic basis of amylase binding where proteins of diverse size and function share a common phenotype.

**Results:**

ABPs were detected in culture supernatants of 27 of 59 strains representing 13 oral *Streptococcus* species screened using the amylase-ligand binding assay. N-terminal sequences from ABPs of diverse size were obtained from 18 strains representing six oral streptococcal species. Genome sequencing and BLAST searches using N-terminal sequences, protein size, and key words identified the gene associated with each ABP. Among the sequenced ABPs, 14 matched amylase-binding protein A (AbpA), 6 matched amylase-binding protein B (AbpB), and 11 unique ABPs were identified as peptidoglycan-binding, glutamine ABC-type transporter, hypothetical, or choline-binding proteins. Alignment and phylogenetic analyses performed to ascertain evolutionary relationships revealed that ABPs cluster into at least six distinct, unrelated families (AbpA, AbpB, and four novel ABPs) with no phylogenetic evidence that one group evolved from another, and no single ancestral gene found within each group. AbpA-like sequences can be divided into five subgroups based on the N-terminal sequences. Comparative genomics focusing on the *abpA* gene locus provides evidence of horizontal gene transfer.

**Conclusion:**

The acquisition of an ABP by oral streptococci provides an interesting example of adaptive evolution.

**Electronic supplementary material:**

The online version of this article (doi:10.1186/s12866-017-1005-7) contains supplementary material, which is available to authorized users.

## Background

The importance of commensal bacteria to the health and wellbeing of the human host is now becoming more recognized. Successful commensals find ways to maintain colonization in challenging environments. In the oral cavity, bacteria are subjected to extremes in pH, the fluctuating availability of nutrients, the stress of salivary flow, and the physical shear forces mediated by chewing and swallowing. Oral bacteria have evolved numerous mechanisms that influence colonization of this environment. One such mechanism is the production of surface proteins that facilitate adhesive interactions with host and bacterial ligands. These interactions often result in enzymatic activity and/or intra- and inter-species signaling functions that help to maintain a relatively stable collection of bacterial species comprising the oral microbiome.

Viridans streptococci are the predominant commensal bacteria colonizing the oral cavity and dental plaque biofilm. These bacteria cluster into five groups (mitis, mutans, anginosus, salivarius and bovis) based on 16S rRNA gene sequencing [1, 2]. Some species within each group, with the exception of bovis, have surface proteins that bind α-amylase [3–6], the predominant enzyme and one of the most abundant components in the saliva of many mammalian species [7–9].

Amylase, together with other salivary components, forms a pellicle that covers all surfaces of the oral cavity [10]. Bacteria have evolved mechanisms to adhere to the salivary pellicle by binding to various components within it, thus providing the substrate for dental plaque formation. Several species of oral streptococci produce cell wall-associated amylase-binding proteins [3, 6, 11]. In addition to mediating the adhesion of bacteria to the salivary pellicle, the binding of host amylase to the bacterial cell surface can facilitate starch metabolism and bacterial growth [12, 13].

Three types of amylase-binding proteins (ABPs) have been formally named, amylase-binding protein A (AbpA) [14], amylase-binding protein B (AbpB) [15], and amylase-binding protein C (AbpC) [16]. AbpA, essential for amylase binding to the streptococcal surface, is a unique protein with no known conserved domains. It is co-transcribed with the downstream sortase B (*srtB*) gene, shown to be essential for covalent attachment of AbpA to the bacterial cell wall [17–19]. AbpB, which is not essential for amylase binding to the bacterial cell surface [15], is homologous to and functions as a dipeptidyl-peptidase [20]. Currently, in the NCBI database, AbpA and AbpB are designated as ‘amylase-binding protein’ and ‘peptidase C69’, respectively. AbpC differs considerably from AbpA and AbpB, and its functional significance remains to be determined [16].

In addition to these three ABPs, oral streptococcal genomes encode other ABPs with molecular weights ranging from 20 to 87 kDa [3]. To date, there has been no formal analysis of the relationship of each of these proteins to each other. We postulate that some or all of these proteins are related and have been acquired by interspecies horizontal gene transfer followed by mutation or recombination. The goal of this study was to therefore investigate how ABPs of oral streptococci are evolutionarily related. Here, using a combination of approaches, we provide evidence of the amylase-binding phenotype among a diverse group of oral streptococci, and the possible acquisition of these genes through horizontal gene transfer. These results provide an interesting example of adaptive evolution.

## Results

### Detection of amylase-binding proteins

**Fig. 1:**
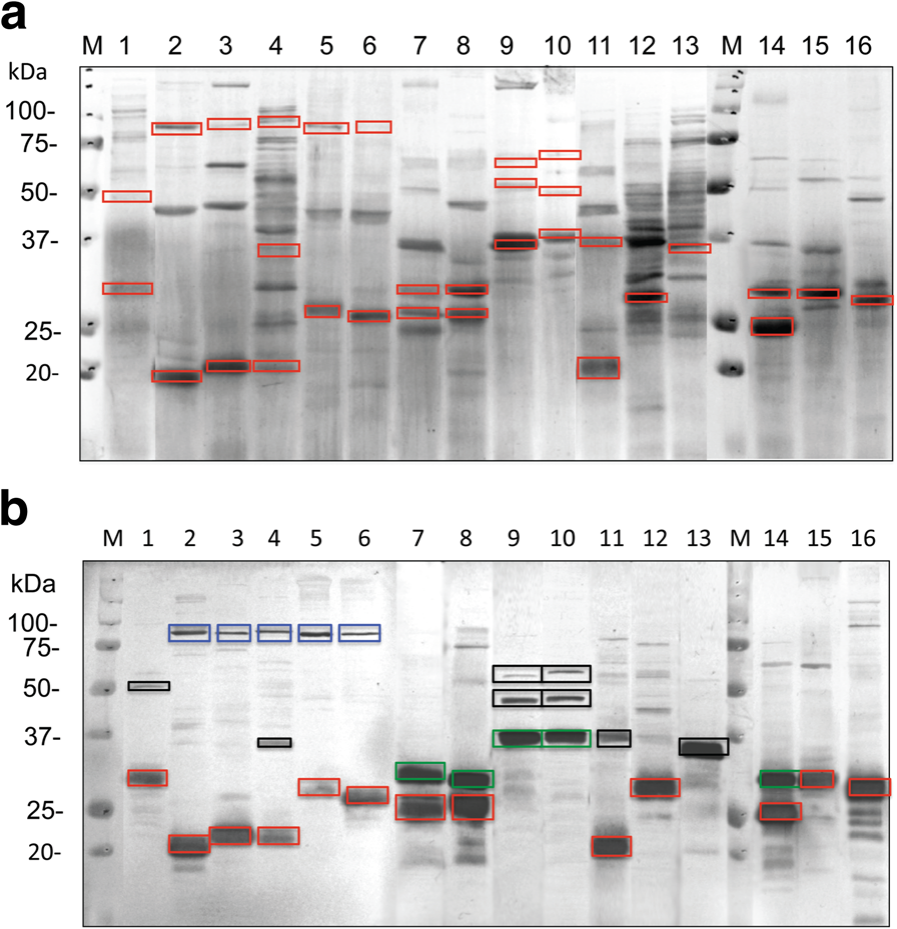
Composite of (**a**) Coomassie-stained gels and (**b**) blots from the amylase-ligand overlay assay. **a** Boxes represent protein bands cut out from the Coomassie-stained blot for N-terminal sequencing. **b** AbpA-like, red boxes; AbpB-like, blue boxes; Novel ABPs, green boxes; Indeterminant, black boxes. Summary of ABPs are listed in Table 2

At the time these experiments were initiated in 2013, few ORFs were annotated as ABPs in the NCBI database. The aim of this study was to search for additional ABPs both in oral streptococcal isolates and in silico. We began by screening 59 oral streptococcal isolates from our collection (Table 1) for the presence of ABPs using the in vitro amylase-ligand binding assay. Although AbpA is initially associated with the cell wall in mid-log phase, by stationary phase AbpA is secreted into the supernatant providing easy access to the native protein. Therefore, AbpA-like proteins and other potential ABPs were obtained from concentrated culture supernatants, resolved by SDS-PAGE, followed by staining with Coomassie blue (Fig. 1a). Proteins from a duplicate gel run at the same time were electrotransferred to polyvinylidene fluoride (PVDF) membrane and assayed for binding to salivary α-amylase in the amylase ligand-binding assay (Fig. 1b). To further characterize these ABPs, selected bands, as listed in Table 2 and outlined by a box in Fig. 1a, were cut from a corresponding Coomassie blue-stained blot (not shown) and sent for N-terminal sequencing. The N-terminal sequence of ABPs from *Streptococcus parasanguinis* MGH413 and *Streptococcus salivarius* KB005 were determined previously (unpublished).

**Table 1:** Amylase binding by streptococcus strains in our collection

Streptococcus species	Strain designation	Amylase ligand-binding overlay^a^ (ca. mw kDa)	Amylase-binding activity^b^	GenBank accession number
*S. anginosus*	ATCC 33397 (NCTC 10713)	-	-	
	UC2953	-	ND	
	UC9218	-	ND	
	NCTC 10708^c^	-	-	
	NCTC 10709^c^	-	-	
*S. australis*	ATCC 700641	22, 35, 86	+	NZ_AFUD01000015
*S. cristatus*	CC5A	26, 26, 84	+	NZ_JYGJ00000000^d.j^
	CR3	28, 28, 84	+	NZ_JYGK00000000^d,j^
	CR311 (ATCC 51100)	30, 82^e^	+	NZ_AFUE01000002
*S. gordonii*	ATCC 10558 (NCTC 7865)	20, 82^e^	ND	
	Blackburn (NCTC 10231)	20, 82^e^	ND	
	Challis CH1	20, 82^e^	+	NC_009785
	I141^i^	20, 84	+	NZ_JYOZ00000000^d^
	CN2814	20, 82^e^	ND	
	FAS4	20, 82^e^	ND	
	GEO2	20, 82^e^	ND	
	G9B	20, 82^e^	+	NZ_JYGL00000000^d,j^
	UB10712^f,i^	20, 37, 82^e^	+	NZ_JYGN00000000^d^
	JF2	20, 82^e^		
	LGR2	20, 82^e^	ND	
	MJ2	20, 82^e^	ND	
	M5	20, 82^e^	ND	
	SPED3	20, 82^e^	ND	
*S. infantis*	ATCC 700779	-	-	PRJNA158721
	UC921A^i^	26, 30	+	NZ_JYGT00000000^d^
	UC6950A^i^	30	+	NZ_JYOV00000000^d^
*S. intermedius*	ATCC 27335 (SK54)	-	-	PRJNA197004
*S. mitis*	NS51/SD142	36 ^g^	+	EF989012.1
	(ATCC 49456)			
	(NCTC 12261)			
	OT25	36	+	NZ_JYGP00000000^d, j^
	SK137	36, 50, 63	+	NZ_JYGQ00000000^d,j^
	SK145	37, 48, 65	+	NZ_JYGS00000000^d,j^
	UC2948	-	-	
	UC3161	-	-	
*S. mutans*	NCTC 10449 (ATCC 25175)	-	-	
	BM71	-	ND	
	GS5	-	ND	
	Ingbritt	-	-	
	LT11	-	ND	
	OMZ175	-	ND	
	NG8	-	ND	
	VT321	-	ND	
	V202	-	ND	
*S. oralis*				
ssp *oralis*	COL85/1862^i^	26, 29	+	NZ_JYGM00000000^d^
ssp *oralis*	OP51^i^	25, 26, 30	+	NZ_JYGO00000000^d^
ssp *oralis*	SK141^h,i^	26, 26, 30	+	NZ_JYGR00000000^d^
ssp *tigurinus*	UC5873^i^	26, 30	+	NZ_JYGU00000000^d^
	ATCC 10557 (NCTC 7864)	-	-	
	BU174	-^e^	ND	
	C104	-	ND	
	CR834	-^e^	ND	
	KS32AR	-	ND	
	MPD1	-^e^	ND	
	SK92	-	-	
	34	-	ND	
	9811	-^e^	ND	
	16532AR	-	ND	
*S. parasanguinis*	MGH413	21, 87	+	NZ_JYOW00000000^d,j^
	VT517^i^	22, 84	+	NZ_JYPA00000000^d^
*S. salivarius*	KB005	29	+	NZ_JYOX00000000^d,j^
	CDC013	29	ND	
	CDC017	29	ND	
	CM103	29	ND	
	MG568	29	ND	
	MG587	29, 51	ND	
	MG691	29, 49	ND	
	UC810	29	ND	
	UC3162	29, 50	+	NZ_JYOY00000000^d^
	TOUR	29	ND	
	13,419-R	29	ND	
*S. sanguinis*	ATCC 10556 (NCTC 7863)	-	-	
	HPC1	-	ND	
	MPC1	-^e^	ND	
	UC9433	-	-	
	804	-	ND	
*S. sobrinus*	B13	-	ND	
	SL1	-	-	
	K1R	-	ND	
	OMZ 176	-	ND	
	6715-WT13	-	ND	

*ND* not done

^a^Amylase-ligand binding assay: detects the ability of proteins from 20× concentrated culture supernatant to bind salivary amylase in whole saliva by Far-Western blot (Methods)

^b^Amylase-binding activity: measures the ability of bacterial cells to bind salivary amylase in whole saliva and reduce hydrolysis in starch agarose. (+) ABP, (-) ABP on bacterial cells (Methods)

^c^ *S. milleri* reclassified as *S. anginosis* in Ruoff et al. [48]

^d^Sabharwal et al. [22]; strains sequenced for this study

^e^Brown et al. [3]

^f^Strain labeled NCTC 10712 (*S. mitis*) in our collection is now corrected to *S. gordonii*, UB10712 upon whole genome sequencing

^g^Vorrasi et al. [16]

^h^The strain from our collection named *S. mitis* in Brown et al. [3] Submitted to GenBank as *S. mitis*; updated to *S. oralis* in 2015 as per reclassification Takenouchi-Ohkubo et al. [28] and Kilian et al. [49]

^i^Updated taxonomic classifications for Mitis group, Jensen et al. [23]

^j^Reference strains used for genome post-assembly: *S. cristatus* ATCC 51100 for *S. cristatus* CC5A and CR3; *S. gordonii* Challis substrain CH1 for *S. gordonii* G9B; *S. mitis* B6 for *S. mitis* OT25, SK145, SK137; *S. parasanguinis* ATCC 15912 for *S. parasanguinis* MGH413; *S. salivarius* CCHSS3 for *S. salivarius* KB005

**Table 2:** Amylase-binding protein summary from Fig. 1b blot

Lane no.	Species	Strain	No. Bands	AbpA-likeca. mw	AbpB-like ca. mw	Novel ca. mw
1	*S. salivarius*	UC3162	2	29^b^		50^c^
2	*S. gordonii*	I141	2	20^b^	84^e^	
3	*S. parasanguinis*	VT517	2	22^b^	84^e^	
4	*S. australis*	ATCC 700641	3	22^e^	86^e^	35^d^
5	*S. cristatus*	CR3	2	28^b^	84^e^	28^b,f^
6	*S. cristatus*	CC5A	2	26^e^	84^e^	26^b,f^
7	*S. oralis*	SK141	2	26^e^		26^b,g^
						30^b,h^
8	*S. infantis*	UC921A	2	26^b^		30^b,h^
9	*S. mitis*	SK137	3	None	None	36^b,i^,50^c^,63^d^
10	*S. mitis*	SK145	3	None	None	37^b,i^,48^d^,65^c^
11	*S. gordonii*	UB10712	2	20^b^	82^e^	37^d^
12	*S. oralis*	COL85/1862	1	29^b^		26^e,g^
13	*S. mitis*	OT25	1	None		36^d^
14	*S. oralis*	OP51	2	25^b^		26^e,g^,30^b,h^
15	*S. oralis*	UC5873	1	30^b^		26^e,g^
16	*S. infantis*	UC6950	1	30^b^		
	*S. parasanguinis* ^*a*^	MGH413	2	21^b^	87^e^	
	*S. cristatus* ^*a*^	NCTC 51100/CR311	2	30^b^	82^e^	26^e,f^
	*S. salivarius* ^*a*^	KB005	1	29^b^		

^a^N-terminal sequences determined previously, not published

^b^Sequence determined based on N-terminal sequence

^c^Insufficient quantity for N-terminal sequencing

^d^Identity indeterminant from N-terminal sequence

^e^Sequence based on whole genome sequence and/or primer walking

^f^Peptidoglycan-binding protein

^g^Glutamine ABC transporter

^h^Hypothetical protein

^i^Choline-binding protein

Several streptococcal isolates expressed more than one ABP. Two ABPs were identified previously in *Streptococcus gordonii* Challis CH1, namely AbpA (20 kDa) and AbpB (82 kDa) [14, 15]. As shown in Fig. 1b, additional ABPs from different strains were identified ranging from 20 to 87 kDa. N-terminal sequencing of all ABPs was cost prohibitive, therefore, proteins were selected to represent a range of molecular weights. Of the 36 protein bands sent for sequencing, 33 bands yielded N-terminal sequences for further in silico analysis. Results from this and a previous study [3] revealed that 41 of 79 (52%) oral streptococcal strains showed the ability to bind amylase by one or more methods (Table 1). Based on these data, none (0/6) of the anginosus group (*S. anginosus*, *S. intermedius*); 64% (30/47) of the mitis group (*S. australis, S. cristatus, S. gordonii, S. infantis, S. mitis*, *S. oralis, S. parasanguinis, S. sanguinis*); none (0/15) of the mutans. group (*S. mutans, S. sobrinus*), and all (11/11) of the salivarius group (*S. salivarius*) were able to bind salivary α-amylase. As previously noted, all *S. gordonii* and no *S. mutans* strains bound amylase. Draft genomes of *S. infantis* strains ATCC 700779 and SK970 do not carry a *abpA* or *srtB* sequence, unlike *S. infantis* strains SK1076, SK1302, UC921A, and UC6950A. Thus, it is not surprising that *S. infantis* ATCC 700779 was negative in the amylase-ligand binding assay. Many *Streptococcus* species were heterogeneous with respect to amylase binding [6]. Of the 59 strains screened, 18 were selected for genome sequencing. The ability of bacterial cells from each of these strains to bind amylase correlated with the amylase binding of their supernatant proteins (Table 1).

### Assembly of whole genome sequencing data

#### Sequenced genomes were assembled de novo

The recently developed algorithm, MyPro [21], facilitated assembly and annotation of the draft genomes. When closely related streptococcal genomes were available for reference, a post-assembly algorithm was used to reduce gaps in the de novo assemblies [21, 22]. Post-assembled genomes consisted of fewer gaps in the assembly, with a median of 6 contigs. De novo assembled genomes ranged in size from 1.79 to 2.29 Mb, with an average G + C content of 40.8% and 1916 coding sequences [22].

### Taxonomic designation

There have been changes in the taxonomic assignment of several mitis group *Streptococcus* species subsequent to the availability of comparative phylogenetic analyses of core genomes, multilocus sequence analysis (MLSA), and 16S rRNA gene sequence data [23]. In the current study, revised streptococcal taxonomy showed changes in species nomenclature of 25 strains used in phylogenetic analyses. Briefly, several members of *mitis*, *oligofermentans*, *pseudopneumoniae*, and *sanguinis* species that represent ABS were revised to other *Streptococcus* species based on MLSA and whole genome core phylogeny [23]. Taxonomic reassignments are summarized in Additional file 1: Table S1, and updates have also been made to our draft genome submissions [22].

### Identification of AbpA-like sequences

The AbpA protein sequence (20–26 kDa) from our newly sequenced strains and homologs obtained in silico were used to further categorize AbpA-like producing streptococcal strains into five subgroups based on the N-terminal sequence. In-depth phylogenetic analysis of AbpA for this study was confined to predominant human oral streptococcal strains and to strains where a sortase B (*srtB*) gene was immediately downstream of the putative *abpA* gene.

Comparison of complete *abpA* gene sequences revealed more heterogeneity among amylase-binding streptococci than previously observed. The *Streptococcus* species and strains within each AbpA-like subgroup along with the associated N-terminal sequences are listed in Additional file 2: Table S2. Subgroup 1 consists primarily of *S. gordonii* strains, but also includes a strain of *S. cristatus.* The well-characterized AbpA protein of *S. gordonii* Challis CH1 is a member of this subgroup [12, 14]. Subgroup 2 is predominantly *S. cristatus* strains, but also contains the Gram-positive opportunistic pathogen *Gemella haemolysans,* a member of the human flora of the oral cavity and upper respiratory tract [24, 25]. Since *G. haemolysans abpA* lacks the typical downstream *srtB* gene, it was excluded from phylogenetic analysis. *S. salivarius* dominates Subgroup 3, but also contains some *S. oralis* and *S parasanguinis* strains. Subgroup 4 is a mix of *S. oralis, S. infantis,* and *S. parasanguinis*. Subgroup 5 is the most diverse group; although *S. parasanguinis* predominates, *S. australis*, S. *infantis*, and *Streptococcus vestibularis* strains are represented among others. The N-terminal sequences not only serve to subgroup AbpA-like proteins, but may contribute to the ability of AbpA to bind amylase, as in *S. gordonii* [19, 26].

**Fig. 2:**
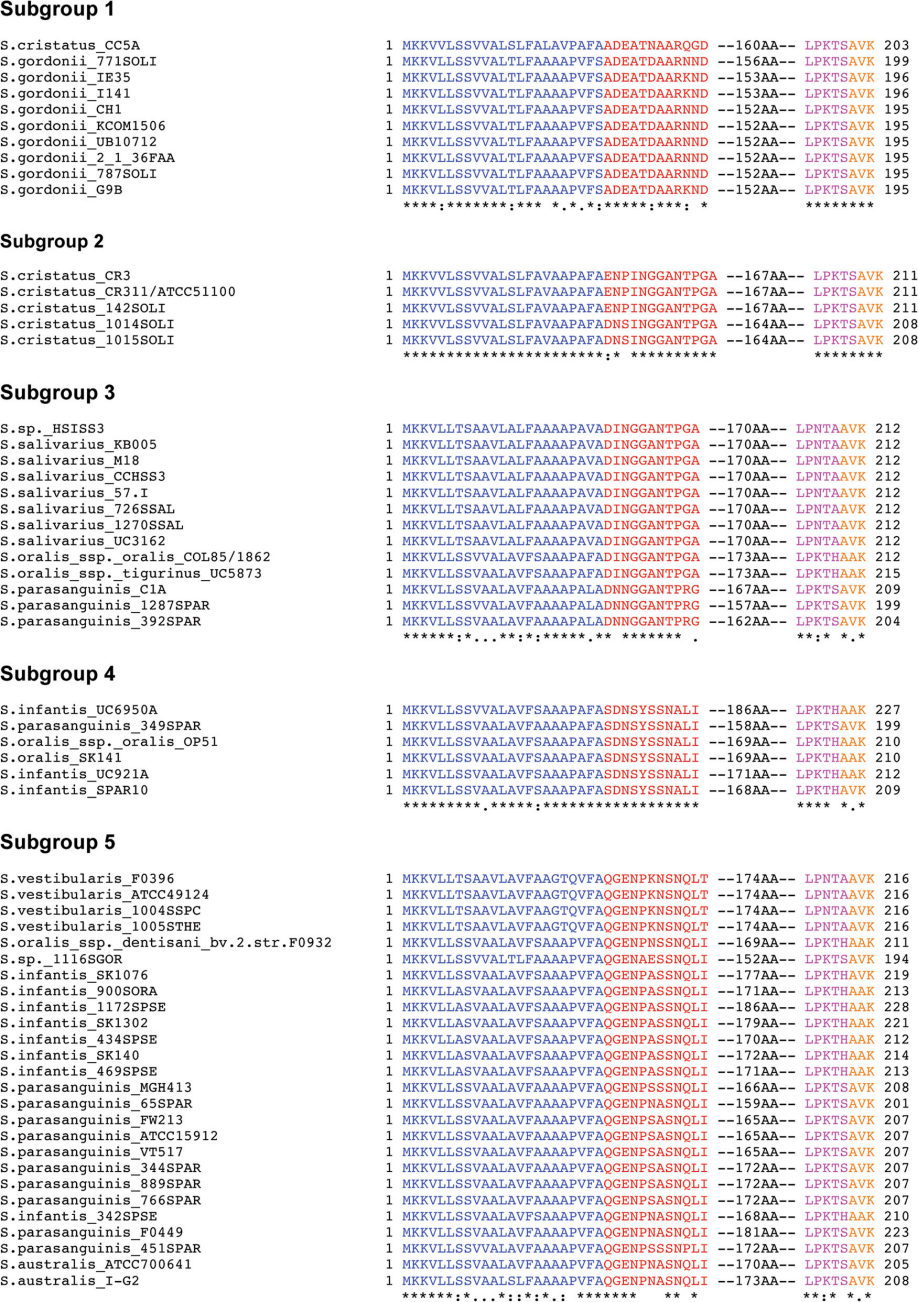
CLUSTAL Omega alignment of functional regions from AbpA-like protein sequences. Similarity in signal sequence (blue), sortase-binding motif (violet) and termination codon (orange). Subgroup (1 to 5) differentiation is based on the N-terminal sequence (red)

**Fig. 3:**
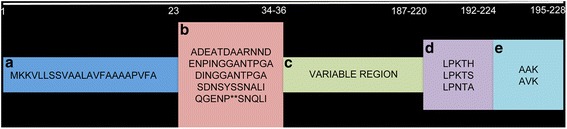
Schematic of AbpA-like proteins. **a** Signal sequence; **b** N-terminal regions for subgroups 1–5; **c** variable region; **d** sortase B-binding motif; and (**e**) termination codon

The AbpA-like protein sequences from each of the subgroups were aligned using CLUSTAL Omega [27]. AbpA sequences of individual subgroup members are well conserved with the exception of subgroup 5, which is the most diverse. When the consensus sequence from each subgroup was aligned, the signal sequence, sortase B-binding motif, and C-terminal end are the most concordant (Fig. 2). These protein sequences contain 195 to 228 amino acid residues and each subgroup revealed a similar arrangement schematically represented in Fig. 3, i.e. a well-conserved signal sequence of 23 residues, followed by one of five subgroup-specific N-terminal consensus sequences (11–13 residues), followed by a variable region of low to moderate homology (152–186 residues), and ending with a putative sortase B-binding motif [LP(K/N)T(S/A/H)] and a termination sequence [A(V/A)K].

### Identification of AbpB-like sequences

Several strains of streptococci produce an 80–87 kDa ABP that is consistent with AbpB (Fig. 1b, Additional file 3: Table S3). Given that the N-terminal amino acid sequences of putative AbpBs were indeterminant due to low protein content in the bands, genomic sequences were instead obtained by primer walking supplemented with NCBI database searches using key words. These data matched a highly conserved group of proteins from the peptidase C69 family (Additional file 4: Figure S1, Additional file 3: Table S3). Previously, a dipeptidyl-peptidase function was confirmed experimentally for AbpB from *S. gordonii* Challis CH1 [20].

### Identification of novel ABPs

Several strains expressing ABPs between 30 and 65 kDa, with N-terminal sequences unlike AbpA or AbpB, were considered novel (Additional file 5: Table S4). Four distinct N-terminal sequences were identified that were consistent with previously described proteins including the LysM superfamily, a peptidoglycan-binding protein; glutamine ABC-type transporter, substrate-binding protein; a hypothetical protein; and a choline-binding protein, cell wall- or glucan-binding domain. Novel proteins were identified in seven of the isolates from this study (CR3, CC5A, SK141, OP51, UC5873, COL85/1862, UC921A), as well as CR311 (ATCC 51100) sequenced earlier. The multiple alignments with other proteins from different *Streptococcus* species obtained from BLAST are presented in Additional file 6: Figure S2; alignments within each group of proteins were well conserved. The AbpC protein from *S. mitis* NS51, identified previously [16], could be aligned with the choline-binding proteins from *S. oralis* strains. Although a glutamine ABC-transporter substrate-binding protein from *Streptococcus pneumoniae* R6 and a choline-binding protein from *S. pneumoniae* SMRU2014 align with novel ABPs, to date no *S. pneumoniae* strains have been shown experimentally to bind salivary amylase.

### Sequence similarity network (SSN)

**Fig. 4:**
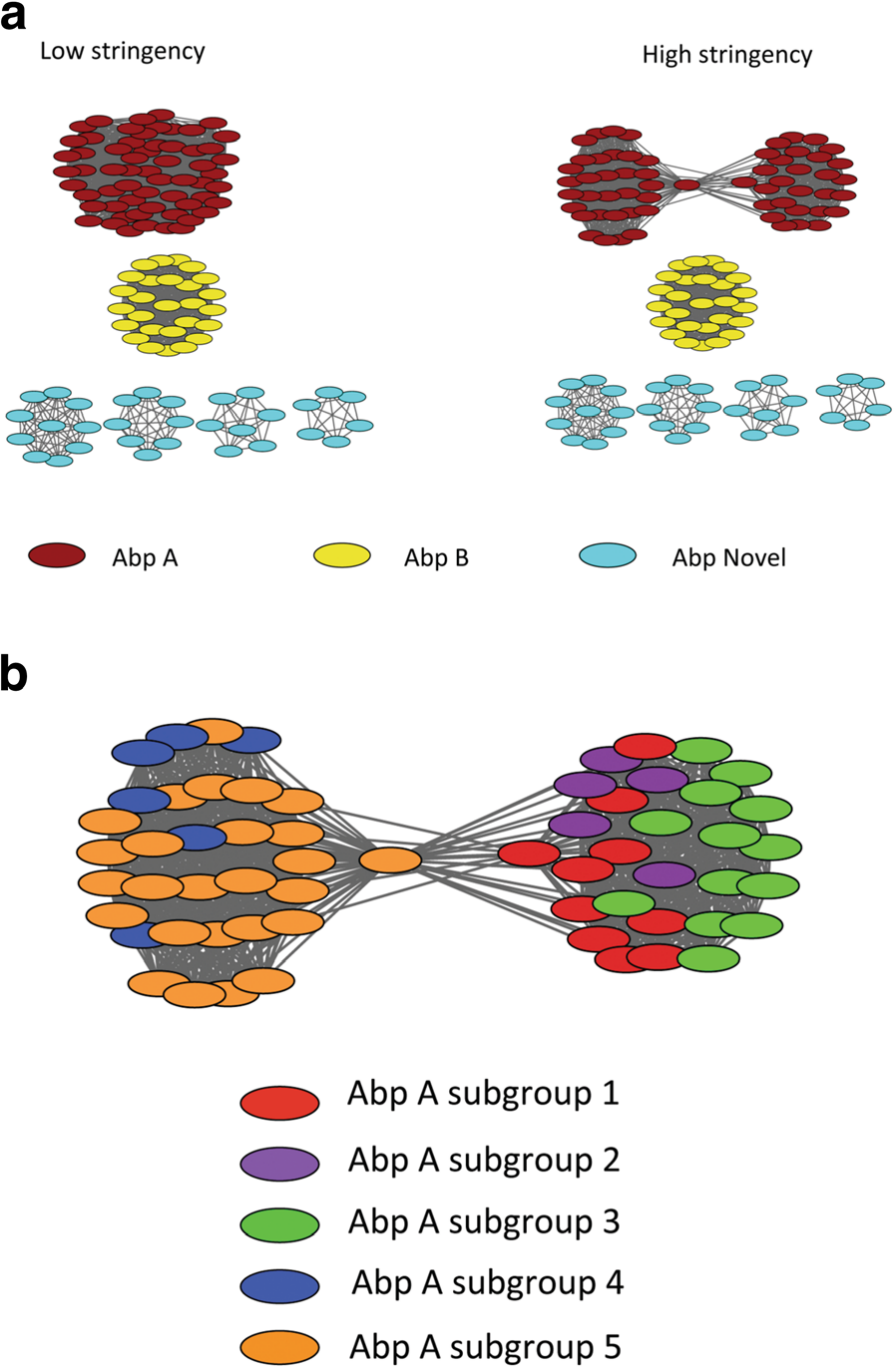
Sequence similarity network of all ABPs. **a** At low stringency, amylase-binding proteins cluster into six families: AbpA (red), AbpB (yellow) and Abp Novel (blue). At high stringency, AbpA separates into two interrelated subfamilies. **b** Sequence similarity network of AbpA subgroups at high stringency. Each AbpA is color-coded according to its classification into one of the five subgroups defined by the N-terminal sequence, a putative amylase-binding motif

**Fig. 5:**
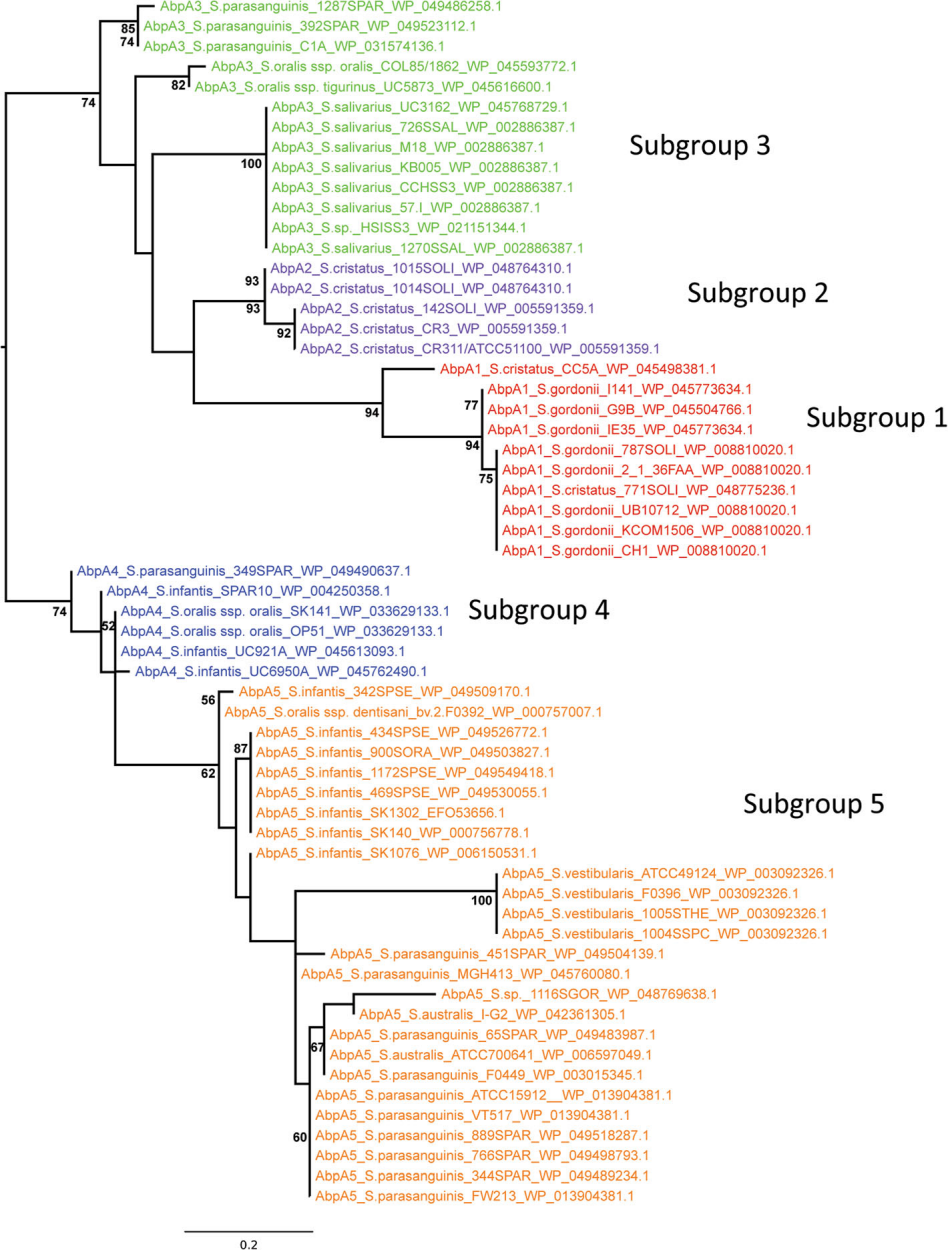
RAxML tree of AbpA subgroups using CLUSTAL W functional regions alignment

Based on the least stringent alignment score, the SSN grouped all AbpAs into one cluster and all AbpBs into another cluster, while novel ABPs were grouped into four separate clusters (Fig. 4a). The distinct clustering among these novel ABP groups suggests that individual clusters represent different gene families. In the high stringency SSN, all AbpAs formed a dumbbell-shaped cluster with a high degree of interconnectivity between the two bells. As shown in Fig. 4b, each AbpA was color-coded according to its classification into one of the five subgroups defined by the N-terminal sequence, a putative amylase-binding motif. The clustering of AbpA subgroups follows the RAxML phylogeny (Fig. 5).

### Phylogenetic analysis of AbpA-like proteins

As shown in Figs. 2 and Additional file 7: Figure S3, the signal sequence, sortase binding motif, and C-terminal regions of the AbpA-like proteins aligned with high reliability, while the N-terminal amylase-binding region was less reliably aligned due to a high degree of sequence divergence. A RAxML analysis based on these functional regions produced a phylogenetic tree that grouped the AbpA subgroups into two major clades receiving moderate bootstrap support (74%), with subgroups 1, 2, and 3 grouping into one clade, and subgroup 4 and 5 grouped into another clade (Fig. 5). However, the interrelationships among subgroups within these two clades are poorly supported, and while subgroups 1, 2, and 5 each receive moderate to strong support, members of subgroup 3 and 4 are non-monophyletic. Subgroup 3 members are split into three well-supported clades following taxonomy (*S. parasanguinis*, *S. oralis*, and *S. salivarius*, respectively). Different PRANK alignments corroborate grouping the AbpA subgroups into the same two major clades (only trees based on complete protein sequences and threshold 7 are shown here; Additional files 8 and 9: Figures S4 and S5). However, a number of incongruent relationships among subgroup members within these two major clades are found. For example, in all trees based on the PRANK alignments, subgroup 3 members now form two clades, and subgroup 4 members are nested within subgroup 5 members, rendering subgroup 5 non-monophyletic. In the analysis based on complete protein sequences, subgroups 1 and 2 are strongly supported as sister groups (Additional file 8: Figure S4), which is also supported by their very similar N-terminal sequences (Figs. 2 and 5). However, in the analyses of trimmed alignments based on PRANK reliability score thresholds, subgroup 2 is either nested within subgroup 3 (Additional file 9: Figure S5) or is sister to subgroup 3 (not shown). Since these phylogenetic trees are unrooted, i.e. reconstructed without outgroup for comparison, conclusions regarding common ancestry are limited. Yet the high degree of overall sequence divergence and relative lack of interrelationships among most subgroups suggest that AbpA-like proteins have evolved independently.

### Evidence of horizontal gene transfer

**Fig. 6:**
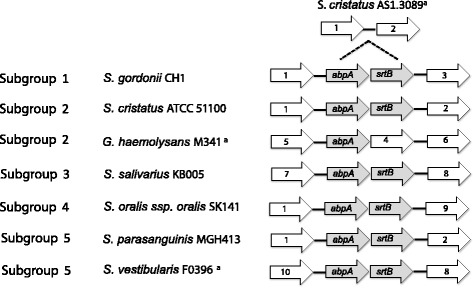
Synteny mapping of *abpA-srtB*. *S. cristatus* AS1.3089 did not carry the *abpA-srtB* locus. ORFs flanking the *abpA-srtB* locus were deduced from genome sequences available at NCBI. All strains except *S. cristatus* AS1.3089, *S. vestibularis* F0396, and *G. haemolysans* M341 have been tested in vitro for the ability to bind amylase. Flanking genes include: (1) ribose-phosphate pyrophosphokinase; (2) aminotransferase; (3) haloacid dehalogenase; (4) MFS transporter; (5) peptidase M42; (6) peptidase; (7) DNA-binding transcriptional regulator, XRE-family; (8) metallophosphatase; (9) CoA-binding protein; and (10) IS200/IS605 family transposase. ^A^ *in silico* evidence only

Complete and nearly complete genomes containing the *abpA-srtB* gene locus were analyzed for evidence of horizontal gene transfer. The G + C content of *abpA* ranged from 38.91 to 42% from genomes with a G + C content ranging from 39.9 to 42.1%. This minimal deviation in G + C content is consistent with ancient genes in closely related species such as the immunoglobulin A1 protease (*iga*) gene from *Streptococcus* species [28]. Synteny maps were constructed (Fig. 6) to examine the immediate gene neighborhood around the *abpA-srtB* gene locus among genomes representative of different AbpA subgroups. There is lack of synteny conservation in genes flanking *abpA-srtB* in *Streptococcus* species experimentally found to bind amylase, in species with in silico evidence of the gene locus, and around the gene encoding the AbpA ortholog in *G. haemolysans* M341. Comparison of *S. cristatus* ATCC 51100 to *S. cristatus* AS 1.3089 demonstrates the presence and absence of *abpA-srtB*, respectively, in closely related strains while preserving the immediate synteny neighborhood. Overall, among ABS species and strains in this study, the genes flanking *abpA-srtB* show conservation within species, but not between species. In addition, transposon-mediated mutagenesis with Tn*916* used to inactivate *abpA* demonstrated a transposon integration hotspot immediately upstream of *abpA* [14]. Together, these observations suggest the co-transcribed *abpA*-*srtB* locus is a genetic island that may have been acquired by horizontal transfer.

### Molecular modeling of AbpA subgroups

**Fig. 7:**
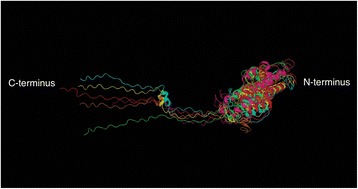
Tertiary protein structure of representative AbpA sequences. N-terminal half of AbpA is predominated by helical spatial arrangement and the C-terminal half is largely coiled. NMR structure of *S. gordonii* CH1 is in red and the predicted structures for representative sequences are coded in various colors (Green-Subgroup1-WP_008810020.1; Yellow-Subgroup2-WP_005591359.1; Purple-Subgroup3-WP_002886387.1; Blue-Subgroup4-WP_033629133.1; Orange-Subgroup5-WP_003092326.1)

In silico modeling with I-TASSER revealed similar tertiary structures for representative AbpA proteins from each subgroup. The molecular modeling performed here is limited by the availability of NMR data on AbpA structure, which until recently was not reported [29]. Overall, helical arrangement predominated the N-terminal half of the molecule and C-terminal half showed a coiled structure (Fig. 7).

## Discussion

In this study, we expand upon previous studies to better understand how oral streptococci use different proteins to bind amylase. This interaction allows the bacteria to adapt to the oral environment by adhering to the salivary pellicle and initiating colonization of the tooth surface, as well as to efficiently metabolize dietary starch as an energy source. An in vitro assay to assess amylase binding to the cell surface of oral *Streptococcus* species has also been used previously in an attempt to classify viridans streptococci [6, 11, 30]. Here, this method was used to confirm the results of amylase binding to denatured proteins in the culture supernatant. Most *Streptococcus* strains examined produced an AbpA-like protein, the best-studied and most predominant protein that mediates the binding of amylase to the bacterial surface. But bacterial cells from four strains, *S. mitis* OT25, SK137, SK145, and NS51 bound amylase without AbpA. These strains produced novel proteins, in particular the AbpC of *S. mitis* NS51. The actual function, cellular location, exposure to the extracellular environment, and relevance of these novel ABPs to the fitness of these strains, remains to be determined.

At least six separate families of ABPs comprising AbpA-like, AbpB-like, and novel proteins were identified in *Streptococcus* strains sequenced in this study in combination with homologs obtained from NCBI RefSeq https://www.ncib.nlm.nih.gov/refseq/. There was no significant similarity between the families based on protein sequence alignment. No readily identifiable amylase-binding or sortase-binding motifs were present except within the AbpA-like family. Thus, binding of amylase by each of these protein families is likely due to secondary structural interaction rather than by primary amino acid sequences. This was especially evident for AbpA-like proteins having moderate amino acid similarity and identity, but with similar predicted secondary structures as presented in this study and by Liang et al. [19].

General functions of ABPs may be in promoting oral colonization and fitness. When ABPs bind salivary amylase, they enable close contact of bacteria with oral surfaces coated with saliva and promote subsequent colonization. When first described, AbpA was a novel protein [14]. Homologs are now found in many oral streptococcal species of humans and animals whose saliva contains amylase activity [32, 33]. In addition to playing a role in adhesion, biofilm formation, providing a convenient source of saccharolytic nutrition through the hydrolysis of dietary starch, and possibly providing relief from oxidative stress, there may be as yet undescribed functions for AbpA [12, 17]. AbpB protein sequences are conserved (63–100% identical), suggesting they have a primary function as dipeptidases, which may have a role in nutrient acquisition pathways [20, 34]. The novel ABPs have been annotated with a variety of cell wall-associated functions. Association of AbpA with the bacterial cell wall, especially at the site of nascent cell wall synthesis, was previously demonstrated [32], so the finding that other unrelated ABPs may also associate with the cell wall is supportive of the hypothesis that amylase binding is beneficial to the growth of the organism [12, 34]. Alternatively, the ability to bind amylase may just be a coincidental function for AbpB and the novel ABPs. How the acquisition of the *abpA*-*srtB* locus or any of the other genes encoding potentially novel ABPs enhances the survival and role of the commensal streptococcal strains as primary colonizers of the oral cavity requires further study.

The ability of a bacterial species to bind salivary amylase allowing it to adapt to the oral environment is not confined to cell wall components of *Streptococcus* species. Fimbria-associated proteins from *S. sanguinis* SK36 [33] and *S. mutans* CS2 [35], a *Streptococcus* species not normally known to bind amylase have been shown to bind amylase. Amylase-binding components have been described in a few other non-streptococcal oral species including a 110-kDa protein of *Fusobacterium nucleatum* subsp. *polymorphum* [36], and the outer membrane lipopolysaccharide of *Aggregatibacter actinomycetemcomitans* [37] and *Porphyromonas gingivalis* [38]. In *A. actinomycetemcomitans,* amylase interferes with bacterial adherence and biofilm formation. Amylases from several sources are able to inhibit the growth of *P. gingivalis* [39]. How the binding of amylase in vivo in a mixed species biofilm enables or hinders interspecies interactions is not known.

Comparative pan-genomic studies provide evidence of genetic exchange with related species sharing the same ecological niche [40, 41]. Our analyses of complete and nearly complete draft genomes of AbpA-encoding oral *Streptococcus* strains revealed that all contained the *abpA*-*srtB* gene locus. Sortase B is essential for initial covalent attachment of AbpA to the bacterial cell wall [18, 19]. In fact this gene locus was found in 15/18 strains sequenced in this study, supporting the role of AbpA as the predominate ABP. The *abpA* gene may be an ancient gene, not unlike another gene in the mitis group of streptococci, *iga*, which encodes IgA protease [28] and is also found in several closely related *Streptococcus* species. AbpA is located in a region of conserved gene order indicating relatively recent subgroup divergence. AbpA subgroups generally correlate with species, although there is evidence of interspecies divergence. For example, within the mitis group of *Streptococcus* species, the *S. oralis* clade is more distantly related to the *S. parasanguinis* clade [23], and both contain strains that carry AbpA-like proteins from a variety of subgroups. This apparent random distribution of the co-transcribed *abpA-srtB* genes in more distantly related *Streptococcus* species, the lack of synteny conservation in closely related species, and the presence of an AbpA ortholog in a different genus (*Gemella*) suggests that these genes are part of a genomic island acquired by horizontal gene transfer.

Many oral streptococci are known to be naturally competent [42], a benefit to organisms living in a biofilm such as dental plaque. While screening our collection of *Streptococcus* strains, it became apparent that not all strains of a given species possess an ABP. The transfer of DNA encoding a beneficial trait from one member of the community to another enhances the survival of the community. It is therefore possible that the acquisition of the amylase-binding function by some oral streptococcal strains, mediated by different proteins enables these bacteria to take advantage of their ecological niche within the oral cavity.

This study demonstrates that ABPs are a diverse group of proteins that sort into at least six distinct groups with a variety of putative functions. Sequence divergence of the ABPs, especially within the AbpA group, suggests that the amylase-binding function evolved independently in separate protein families. The ability to bind amylase may benefit the host by serving to remove bacteria in saliva. The high prevalence of amylase binding among primary colonizing streptococci in dental plaque may demonstrate how adaptive evolution can enhance bacterial colonization and survival in the oral cavity. Amylase binding may not only facilitate the nutrition of bacteria in the immediate environment, but it may also serve another unrecognized function to benefit the whole microbial community.

## Conclusions

In this study, we explored the genomic and evolutionary aspects of ABPs. We demonstrated that ABPs (AbpA, AbpB and novel ABPs) cluster into six distinct but unrelated families. Further, the AbpA protein family could be subgrouped based on N-terminal sequences. While evidence for evolution of these protein families was not conclusive, comparative genomics of *abpA* gene provided evidence of horizontal gene transfer. Importantly, the acquisition of ABP by oral streptococci provided an interesting example of how bacteria may evolve to adapt to the human host.

## Methods

### Bacterial strains and culture conditions

A total of 79 oral streptococcal strains representing 13 species were screened for ABPs, which included 20 strains studied previously and 59 strains examined here (Table 1). All strains were obtained from our culture collection, with the exception of *S. australis* ATCC 700641 and *S. infantis* ATCC 700779, which were obtained from the American Type Culture Collection (ATCC, Manassas, VA). Bacteria were cultured from frozen stocks to agar plates containing tryptic soy broth supplemented with 0.5% (*w*/*v*) yeast extract (TSBY) and 1.5% Bacto agar (Becton Dickinson and Co., Sparks, MD) in a candle jar at 37 °C for 2 days. Isolated colonies were subcultured into TSBY broth and incubated in a candle jar at 37 °C for 14 to 16 h.

### Human saliva collection

The University at Buffalo Human Subjects Institutional Review Board approved the saliva collection protocol. Briefly, unstimulated whole saliva, as the source of human salivary amylase, was collected from several donors by expectoration into ice-chilled tubes, clarified by centrifugation at 12,800 x *g* for 10 min at 4 °C, aliquoted, and stored at −20 °C until use.

### Detection of amylase-binding proteins (ABPs) by the amylase ligand-binding assay

Streptococcal culture supernatants from overnight cultures were collected by centrifugation at 5000×*g* for 10 min followed by 20-fold concentration using Amicon Ultra-4 centrifugal filter devices (Merck Millipore Ltd., Tullagreen, Carrigtwohill, Co. Cork, IRL). The concentrated supernatant proteins were separated on a 12.5% gel by SDS-PAGE. Proteins were either stained with Coomassie blue or transblotted to PVDF membrane. The amylase-ligand binding assay was performed as previously described [14, 20]. Whole human saliva was used as the source of amylase.

### N-terminal amino acid sequencing

Concentrated supernatants containing ABPs identified by the aforementioned amylase ligand-binding assay were subjected to 12.5% SDS-PAGE and separated proteins were electrotransferred from gels to a small pore PVDF ProBlott membrane (Applied Biosystems Inc., Foster City, CA) and stained with 0.1% (*w*/*v*) Coomassie brilliant blue R-250. The protein bands corresponding to the identified ABPs were cut from the blot and N-terminal sequenced by standard methods (ProSeq Inc., Oxford, MA). The N-terminal amino acid sequences of ABPs were used to search for homologous proteins in the National Center for Biotechnology Information database (NCBI, http://www.ncbi.nlm.nih.gov).

### Purification and sequencing of streptococcal DNA

Genomic DNA isolated from streptococcal strains using a previously described method [20] was used as the template in PCR reactions. For high-throughput sequencing, the genomic DNA was first treated with RNase A/T1 Mix (Thermo Fisher Scientific Inc., Pittsburgh, PA) according to the manufacturers’ instructions. The QIAamp DNA Mini Kit (Qiagen, Hilden, Germany) was then used to further purify the genomic DNA. The quality was assessed by ethidium bromide stained agarose gels. DNA was quantified at A_260_ and A_280_ using the Nanodrop spectrophotometer and the Quant-iT dsDNA kit (Invitrogen, Carlsbad, CA). Samples were stored at −80 °C prior to library construction for whole genome sequencing (Center for Excellence in Bioinformatics at the University at Buffalo, Buffalo, NY).

### Sequencing, quality control, de novo assembly, and annotation of streptococcal genomes

Illumina HiSeq 2500 Next-Generation Sequencing was used in rapid 150-cycle paired-end mode to perform genome sequencing of the appropriately prepared streptococcal strains. The paired-end sequencing reads of 150-bp read length and over 100X coverage were processed using MyPro (http://sourceforge.net/projects/sb2nhri/files/MyPro) [21], a customized software pipeline designed for prokaryotic genome assembly and annotation. Quality control tests, de novo assembly, and annotation were applied to genomes of 18 streptococcal strains. When closely related reference genomes were available, post-assembly function of MyPro was used to align and order contigs and reduce gaps in the de novo genome assemblies [22]. All draft genome sequences were deposited in GenBank and corresponding accession numbers were published prior to data mining [22].

### Identification of amylase-binding protein a sequences

Complete *abpA* were manually obtained from 18 streptococcal genomes by using corresponding N-terminal sequence as a query. Thereafter, complete *abpA* sequences were used as multifasta query to BLAST against the non-redundant protein sequence (nr) database restricted to Streptococcus (taxid:1301) using an evalue of 1e-5. Results were curated for presence of N-terminal - like sequences as a measure of confirmation.

### Identification of amylase-binding protein B sequences

To obtain genomic sequences previously annotated as AbpB, the NCBI RefSeq database was searched for complete streptococcal AbpB sequences by using the keywords ‘amylase-binding protein’ or ‘amylase-binding protein B’, and filters ‘bacteria’ and ‘sequence length more than 300’. The results were un-collapsed and manually sorted to include all non-redundant streptococcal strains. To identify any additional AbpB-like sequences that may be annotated differently (e. g. peptidase C69), the database was also searched with sequences obtained from primer walking of AbpB.

### Primer walking of *abpB*

To determine the *abpB* sequence from each of the strains expressing AbpB in the amylase-ligand binding assay, five sets of degenerate primers were designed according to the alignment of nucleotide sequences of known AbpBs. Primers were designed to conserved regions where possible. The primer sequences (Invitrogen) listed in Additional file 10: Table S5 cover approximately 84% of *abpB*. Conventional PCR was initiated with 90 s at 95 °C, followed by 34 cycles of 30 s at 95 °C, 30 s at 50–52 °C according to the annealing temperatures, and 2 min at 72 °C, then 10 min at 72 °C using a T100 thermo cycler (Bio-Rad, CA). Amplified products were separated in a 1.5% agarose gel, stained with ethidium bromide, and visualized under UV light. Genomic DNA of *S. gordonii* Challis CH1 and a no template control were used as the positive and negative controls, respectively. PCR amplicons purified by QIAquick PCR Purification kit or QIAquick Gel Extraction kit (Qiagen, Hilden, Germany) were sent for nucleic acid sequencing (Roswell Park Cancer Institute, Buffalo, NY). Sequences obtained from each strain were assembled using SeqMan software (www.dnastar.com) into one sequence containing the nearly complete *abpB* gene.

### Identification of novel ABP sequences

Proteins that bound amylase in the amylase ligand-binding assay with molecular weights ranging from 30 to 65 kDa and whose N-terminal sequences were not similar to one of the five AbpA, or AbpB N-terminal sequences were considered novel ABPs. The NCBI RefSeq database was searched with the N-terminal sequences of these strains to identify novel ABP sequences.

### Sequence similarity network

A sequence similarity network (SSN) of all the ABP protein sequences was generated using EFI-EST [43]. The relatedness is described by sequence similarity based on pairwise alignment scores calculated from an all-by-all BLAST with a default E-value 1e-05. Networks were generated based on the minimum alignment score 6 (low stringency) and the maximum alignment score 16 (high stringency), respectively. Each node, which represents one protein sequence, was color-coded according to its classification in the three ABP groups: AbpA, AbpB, and Abp Novel.

### Phylogenetic analysis of ABPs

Multiple sequence alignment was performed using CLUSTAL W to align the functional regions of AbpA, including the signal sequence, the N-terminal amylase-binding region, the sortase B-binding motif, and the C-terminus. Maximum Likelihood (ML) analysis was performed on the resulting alignment using the RAxML (Randomized Accelerated Maximum Likelihood) algorithm [31]. Since the AbpA-like protein sequences are highly divergent, we also applied PRANK (Probabilistic Alignment Kit) [44] to align the complete protein sequences of AbpAs, and the reliability score of each aligned site was then annotated. The scale of the reliability score is defined as 0 to 9, indicating less reliably aligned site to more reliably aligned site. The alignment was then trimmed based on different reliability score thresholds: 1, 4, 5, 6, 7 and 9. ML analysis using RAxML was performed for each of these trimmed alignments, as well as the complete protein sequences.

### Bacterial amylase-binding assay

The starch agarose assay was performed as previously [6, 11] to access the ability of bacterial cells to bind amylase in clarified whole human saliva. Control samples included saliva alone, and saliva pre-incubated with streptococcal strains known to bind amylase or unable to bind amylase.

### Signatures of horizontal gene transfer surrounding *abpA*

GenBank files for ABS were compared for synteny (one gene upstream and downstream) flanking the *abpA-srtB* locus. Representative genomes for each AbpA subgroup were selected based on experimental evidence of AbpA to illustrate the variation in synteny neighborhood of *abpA-srtB* locus. The closely related genome *S. cristatus* ATCC 51100 was selected based on the lack of the *abpA-srtB* locus, and the *G. haemolysans* M341 genome was selected to illustrate an *abpA* ortholog without the typical *srtB*.

### Molecular modeling of AbpA

Multispecies sequences (representing 20 sequences) from all AbpA subgroups were submitted for molecular modeling using the I-TASSER (Iterative Threading ASSEmbly Refinement) web service, which uses a hierarchical approach for protein structure and function prediction [45]. We included the AbpA sequence from *S. gordonii* Challis CH1 as a positive control. The NMR structure of AbpA from *S. gordonii* G9B has been determined [29], and the protein sequence of *S. gordonii* Challis CH1 differs from G9B only at residue 33 containing Asn and Lys, respectively. I-TASSER predicted secondary structures and tertiary structure models (.pdb format) were obtained for all sequences along with their confidence scores. Model with highest confidence score for each subgroup were used as input for Protean3D (http://www.dnastar.com/t-protean-3D.aspx) for structural comparison and output in graphical format.

### Streptococcal taxonomy

Concatemers of seven housekeeping genes for viridans group Streptococci in multifasta format were obtained from the authors of a previous publication [46]. These sequences were curated to represent the amylase-binding streptococcus (ABS) species used in this study. Curated sequences were used as query to BLAST against a local database comprised of whole genome sequences of ABS with an Expected (E) value of 1e-5 [47]. The results were concatenated in-frame, in the order (*map*-*pfl*-*ppaC*-*pyk*-*rpoB*-*sodA*-*tuf*) and used for phylogenetic analysis. ABS concatemers were aligned using CLUSTAL W of the MEGA 7.0.18 package and the aligned sequences were used to construct a minimum evolution tree.

## Additional files

Additional file 1: Table S1. MLSA based revised streptococcal taxonomy. Includes the genome accession number, strain identifier, old taxonomic name, and new taxonomic name for each strain. (DOCX 91 kb)

Additional file 2: Table S2. Amylase-binding protein A-like subgroup comparison. Includes NCBI protein identifiers, molecular weights, and N-terminal sequences. (DOCX 107 kb)

Additional file 3: Table S3. Amylase-binding protein B-like comparison. Includes NCBI protein identifiers, molecular weights, and N-terminal sequences. (DOCX 89 kb)

Additional file 4: Figure S1. CLUSTAL alignment of AbpB-like protein sequences. (DOCX 167 kb)

Additional file 5: Table S4. Novel amylase-binding protein comparison. Includes NCBI protein identifiers, molecular weights, and N-terminal sequences. (DOCX 82 kb)

Additional file 6: Figure S2. CLUSTAL alignment of novel amylase-binding proteins. (DOCX 145 kb)

Additional file 7: Figure S3. PRANK reliability annotation of the alignment of functional regions (signal sequence and N-terminal sequence) from AbpA-like proteins. (TIFF 11836 kb)

Additional file 8: Figure S4. PRANK tree of AbpA subgroups using the entire gene sequence. (TIFF 14602 kb)

Additional file 9: Figure S5. PRANK tree of AbpA subgroups using the entire gene sequence, reliability score 7. (TIFF 14758 kb)

Additional file 10: Table S5. Degenerate PCR primers for genes encoding AbpB-like proteins. (DOCX 73 kb)

## Abbreviations

AbpA: Amylase-binding protein A
AbpB: Amylase-binding protein B
AbpC: Amylase-binding protein C
ABPs: Amylase-Binding Proteins
ATCC: American Type Culture Collection
BLAST: Basic Local Alignment Search Tool
CLUSTAL W: CLUSTer ALignment W
G + C: Guanine and Cytosine
IgA: Immunoglobulin A1 protease
*iga*: Immunoglobulin A1 protease gene
I-TASSER: Iterative Threading ASSEmbly Refinement
kDa: kilo Dalton
MLSA: MultiLocus Sequence Analysis
NCBI: National Center for Biotechnology Information
NMR: Nuclear Magnetic Resonance
PRANK: PRobabilistic AligNment Kit
PVDF: PolyVinyliDene Fluoride
RAxML: Randomized Accelerated Maximum Likelihood
SDS-PAGE: Sodium Dodecyl Sulfate- PolyAcrylamide Gel Electrophoresis
*srtB*: Sortase B gene
SrtB: Sortase B protein
SSN: Sequence Similarity Network
TSBY: Tryptic Soy Broth supplemented with 0.5% (*w*/*v*) Yeast extract
