# Erratum to: comparative genomics and evolution of the amylase-binding proteins of oral streptococci

**DOI:** 10.1186/s12866-017-1060-0

**Published:** 2017-07-03

**Authors:** Elaine M. Haase, Yurong Kou, Amarpreet Sabharwal, Yu-Chieh Liao, Tianying Lan, Charlotte Lindqvist, Frank A. Scannapieco

**Affiliations:** 1 Department of Oral Biology, School of Dental Medicine, University at Buffalo, State University of New York, Buffalo, NY USA; 2 Division of Biostatistics and Bioinformatics, Institute of Population Health Sciences, National Health Research Institutes, Miaoli, Taiwan; 3 Department of Biological Sciences, University at Buffalo, State University of New York, Buffalo, NY USA; 4 Department of Oral Biology, School of Stomatology, China Medical University, Shenyang, People’s Republic of China

## Erratum

Upon publication of the original article [1] discrepancies were highlighted in the references of this manuscript. On page 11, the references in the following sentence should be amended *“Homologs are now found in many oral streptococcal species of humans and animals whose saliva contains amylase activity [32, 33].”* Citation 33 should read 34 and was incorrectly amended during the proofing process. In addition, an updated reference list has been included below as two references were accidentally omitted.

## Updated reference list

 1. Bentley RW, Leigh JA, Collins MD. Intrageneric structure of *Streptococcus* based on comparative analysis of small-subunit rRNA sequences. Int J Syst Bacteriol. 1991;41(4):487–94. doi:10.1099/00207713%E2%80%9341%E2%80%934-487.

2. Kawamura Y, Hou XG, Sultana F, Miura H, Ezaki T. Determination of 16S rRNA sequences of *Streptococcus mitis* and *Streptococcus gordonii* and phylogenetic relationships among members of the genus *Streptococcus*. Int J Syst Bacteriol. 1995;45(2):406–8. doi:10.1099/00207713%E2%80%9345%E2%80%932-406.

3. Brown AE, Rogers JD, Haase EM, Zelasko PM, Scannapieco FA. Prevalence of the amylase-binding protein A gene (*abpA*) in oral streptococci. J Clin Microbiol. 1999;37(12):4081–5.

4. Douglas CW, Heath J, Hampton KK, Preston FE. Identity of viridans streptococci isolated from cases of infective endocarditis. J Med Microbiol. 1993;39(3):179–82. doi:10.1099/00222615%E2%80%9339%E2%80%933-179.

5. Gwynn JP, Douglas CW. Comparison of amylase-binding proteins in oral streptococci. FEMS Microbiol Lett. 1994;124(3):373–9.

6. Kilian M, Nyvad B. Ability to bind salivary alpha-amylase discriminates certain viridans group streptococcal species. J Clin Microbiol. 1990;28(11):2576–7.

7. Karn RC, Shulkin JD, Merritt AD, Newell RC. Evidence for post-transcriptional modification of human salivary amylase (amyl) isozymes. Biochem Genet. 1973;10(4):341–50.

8. Boehlke C, Zierau O, Hannig C. Salivary amylase - The enzyme of unspecialized euryphagous animals. Arch Oral Biol. 2015;60(8):1162–76. doi:10.1016/j.archoralbio.2015.05.008.

9. Aguirre A, Levine MJ, Cohen RE, Tabak LA. Immunochemical quantitation of alpha-amylase and secretory IgA in parotid saliva from people of various ages. Arch Oral Biol. 1987;32(4):297–301.

10. Scannapieco FA, Torres G, Levine MJ. Salivary alpha-amylase: role in dental plaque and caries formation. Crit Rev. Oral Biol Med. 1993;4(3–4):301–7.

11. Douglas CW. The binding of human salivary alpha-amylase by oral strains of streptococcal bacteria. Arch Oral Biol. 1983;28(7):567–73.

12. Rogers JD, Palmer RJ, Jr., Kolenbrander PE, Scannapieco FA. Role of *Streptococcus gordonii* amylase-binding protein A in adhesion to hydroxyapatite, starch metabolism, and biofilm formation. Infect Immun. 2001;69(11):7046–56.

13. Nikitkova AE, Haase EM, Vickerman MM, Gill SR, Scannapieco FA. Response of fatty acid synthesis genes to the binding of human salivary amylase by *Streptococcus gordonii*. Appl Environ Microbiol. 2012;78(6):1865–75. doi:10.1128/AEM.07071-11.

14. Rogers JD, Haase EM, Brown AE, Douglas CW, Gwynn JP, Scannapieco FA. Identification and analysis of a gene (*abpA*) encoding a major amylase-binding protein in *Streptococcus gordonii*. Microbiology. 1998;144 (Pt 5):1223–33.

15. Li L, Tanzer JM, Scannapieco FA. Identification and analysis of the amylase-binding protein B (AbpB) and gene (*abpB*) from *Streptococcus gordonii*. FEMS Microbiol Lett. 2002;212(2):151–7.

16. Vorrasi J, Chaudhuri B, Haase EM, Scannapieco FA. Identification and characterization of amylase-binding protein C from *Streptococcus mitis* NS51. Mol Oral Microbiol. 2010;25(2):150–6. doi:10.1111/j.2041-1014.2009.00554.x.

17. Haase EM, Feng X, Pan J, Miecznikowski JC, Scannapieco FA. Dynamics of the *Streptococcus gordonii* transcriptome in response to media composition, salivary alpha-amylase and starch. Appl Environ Microbiol. 2015. doi:10.1128/AEM.01221-15.

18. Liang X, Chen Y, Wu H. Sortase B assemble amylase-binding protein A to streptococcal cell surface. 89th Gen. Sess. of Internat. Assoc. Dent. Res.; San Diego, CA: IADR; 2011. p. 72.

19. Liang X, Liu B, Zhu F, Scannapieco FA, Haase EM, Matthews S et al. A distinct sortase SrtB anchors and processes a streptococcal adhesin AbpA with a novel structural property. Sci Rep. 2016;6:30,966. doi:10.1038/srep30966.

20. Chaudhuri B, Paju S, Haase EM, Vickerman MM, Tanzer JM, Scannapieco FA. Amylase-binding protein B of *Streptococcus gordonii* is an extracellular dipeptidyl-peptidase. Infect Immun. 2008;76(10):4530–7. doi:10.1128/IAI.00186-08.

21. Liao YC, Lin HH, Sabharwal A, Haase EM, Scannapieco FA. MyPro: A seamless pipeline for automated prokaryotic genome assembly and annotation. J Microbiol Methods. 2015;113:72–4. doi:10.1016/j.mimet.2015.04.006.

22. Sabharwal A, Liao YC, Lin HH, Haase EM, Scannapieco FA. Draft genome sequences of 18 oral streptococcus strains that encode amylase-binding proteins. Genome Announc. 2015;3(3):*e00510–15* doi:10.1128/genomeA.00510-15.

23. Jensen A, Scholz CF, Kilian M. Re-evaluation of the taxonomy of the Mitis group of the genus *Streptococcus* based on whole genome phylogenetic analyses, and proposed reclassification of *Streptococcus dentisani* as *Streptococcus oralis* subsp. *dentisani* comb. nov., *Streptococcus tigurinus* as *Streptococcus oralis* subsp. *tigurinus* comb. nov., and *Streptococcus oligofermentans* as a later synonym of *Streptococcus cristatus*. Int J Syst Evol Microbiol. 2016;66(11):4803–20. doi:10.1099/ijsem.0.001433.

24. Facklam R, Elliott JA. Identification, classification, and clinical relevance of catalase-negative, gram-positive cocci, excluding the streptococci and enterococci. Clin Microbiol Rev. 1995;8(4):479–95.

25. Collins M. The Genus Gemella. In: Dworkin M, Falkow S, Rosenberg E, Schleifer H, Stackebrandt E, editors. The Prokaryotes. New York: Springer; 2006. p. 511–8.

26. Gopal P, Ragunath C, Vyas V, Shanmugam M, Ramasubbu N. Probing the interaction of human salivary alpha-amylase and amylase binding protein A (AbpA) of *Streptococcus gordonii*. Mol Biol. 2013;2:111. doi:10.4172/2168%E2%80%939547.100011.

27. Sievers F, Wilm A, Dineen D, Gibson TJ, Karplus K, Li W et al. Fast, scalable generation of high-quality protein multiple sequence alignments using CLUSTAL Omega. Mol Syst Biol. 2011;7:539. doi:10.1038/msb.2011.75.

28. Takenouchi-Ohkubo N, Mortensen LM, Drasbek KR, Kilian M, Poulsen K. Horizontal transfer of the immunoglobulin A1 protease gene (*iga*) from *Streptococcus* to *Gemella haemolysans*. Microbiology. 2006;152(Pt 7):2171–80. doi:10.1099/mic.0.28801-0.

29. Sethi A, Mohanty B, Ramasubbu N, Gooley PR. Structure of amylase-binding protein A of *Streptococcus gordonii*: a potential receptor for human salivary alpha-amylase enzyme. Protein Sci. 2015;24(6):1013–8. doi:10.1002/pro.2671.

30. Douglas CW, Pease AA, Whiley RA. Amylase-binding as a discriminator among oral streptococci. FEMS Microbiol Lett. 1990;54(1–3):193–7.

31. Stamatakis A. RAxML version 8: a tool for phylogenetic analysis and post-analysis of large phylogenies. Bioinformatics. 2014;30(9):1312–3. doi:10.1093/bioinformatics/btu033.

32. Scannapieco FA, Haraszthy GG, Cho MI, Levine MJ. Characterization of an amylase-binding component of *Streptococcus gordonii* G9B. Infect Immun. 1992;60(11):4726–33.

33. Okahashi N, Nakata M, Terao Y, Isoda R, Sakurai A, Sumitomo T et al. Pili of oral *Streptococcus sanguinis* bind to salivary amylase and promote the biofilm formation. Microb Pathog. 2011;50(3–4):148–54. doi:10.1016/j.micpath.2011.01.005.

34. Nikitkova AE, Haase EM, Scannapieco FA. Taking the starch out of oral biofilm formation: molecular basis and functional significance of salivary alpha-amylase binding to oral streptococci. Appl Environ Microbiol. 2013;79(2):416–23. doi:10.1128/AEM.02581-12.

35. Perrone M, Gfell LE, Fontana M, Gregory RL. Antigenic characterization of fimbria preparations from *Streptococcus mutans* isolates from caries-free and caries-susceptible subjects. Clin Diagn Lab Immunol. 1997;4(3):291–6.

36. Zulfiqar M, Yamaguchi T, Sato S, Oho T. Oral *Fusobacterium nucleatum* subsp. *polymorphum* binds to human salivary alpha-amylase. Mol Oral Microbiol. 2013;28(6):425–34. doi:10.1111/omi.12036.

37. Baik JE, Hong SW, Choi S, Jeon JH, Park OJ, Cho K et al. Alpha-amylase is a human salivary protein with affinity to lipopolysaccharide of *Aggregatibacter actinomycetemcomitans*. Mol Oral Microbiol. 2013;28(2):142–53. doi:10.1111/omi.12011.

38. Choi S, Baik JE, Jeon JH, Cho K, Seo DG, Kum KY et al. Identification of *Porphyromonas gingivalis* lipopolysaccharide-binding proteins in human saliva. Molecular immunology. 2011;48(15–16):2207–13. doi:10.1016/j.molimm.2011.06.434.

39. Ochiai A, Harada K, Hashimoto K, Shibata K, Ishiyama Y, Mitsui T et al. alpha-Amylase is a potential growth inhibitor of *Porphyromonas gingivalis*, a periodontal pathogenic bacterium. J Periodontal Res. 2014;49(1):62–8. doi:10.1111/jre.12079.

40. Donati C, Hiller NL, Tettelin H, Muzzi A, Croucher NJ, Angiuoli SV et al. Structure and dynamics of the pan-genome of *Streptococcus pneumoniae* and closely related species. Genome Biol. 2010;11(10):R107. doi:10.1186/gb-2010-11-10-r107.

41. Richards VP, Palmer SR, Pavinski Bitar PD, Qin X, Weinstock GM, Highlander SK et al. Phylogenomics and the dynamic genome evolution of the genus *Streptococcus*. Genome Biol Evol. 2014;6(4):741–53. doi:10.1093/gbe/evu048.

42. Cvitkovitch DG. Genetic competence and transformation in oral streptococci. Crit Rev. Oral Biol Med. 2001;12(3):217–43.

43. Atkinson HJ, Morris JH, Ferrin TE, Babbitt PC. Using sequence similarity networks for visualization of relationships across diverse protein superfamilies. PLoS One. 2009;4(2):e4345. doi:10.1371/journal.pone.0004345.

44. Loytynoja A, Goldman N. webPRANK: a phylogeny-aware multiple sequence aligner with interactive alignment browser. BMC Bioinformatics. 2010;11:579. doi:10.1186/1471%E2%80%932105%E2%80%9311-579.

45. Zhang Y. I-TASSER server for protein 3D structure prediction. BMC Bioinformatics. 2008;9:40. doi:10.1186/1471%E2%80%932105%E2%80%939-40.

46. Bishop CJ, Aanensen DM, Jordan GE, Kilian M, Hanage WP, Spratt BG. Assigning strains to bacterial species via the internet. BMC Biol. 2009;7:3. doi:10.1186/1741%E2%80%937007%E2%80%937-3.

47. Altschul SF, Gish W, Miller W, Myers EW, Lipman DJ. Basic local alignment search tool. J Mol Biol. 1990;215(3):403–10. doi:10.1016/S0022-2836(05)80360-2.

48. Ruoff KL. *Streptococcus anginosus* (“*Streptococcus milleri*”): the unrecognized pathogen. Clin Microbiol Rev. 1988;1(1):102–8.

49. Kilian M, Poulsen K, Blomqvist T, Havarstein LS, Bek-Thomsen M, Tettelin H et al. Evolution of *Streptococcus pneumonia*e and its close commensal relatives. PLoS One. 2008;3(7):e2683. doi:10.1371/journal.pone.0002683.
