# Supplementary material for: Comparative genomics and evolution of the amylase-binding proteins of oral streptococci
Source: BMC Microbiol. 2017 Apr 20;17:94. doi: 10.1186/s12866-017-1005-7 (PMC5399409; doi:10.1186/s12866-017-1005-7)
Supplement: Supplementary file 1 — MLSA based revised streptococcal taxonomy. Includes the genome accession number, strain identifier, old taxonomic name, and new taxonomic name for each strain. (DOCX 91 kb) [file 12866_2017_1005_MOESM1_ESM.docx]

**Table S1** MLSA based revised streptococcal taxonomy

| Genome accession  number | Strain | | Old designation | | Revised  designation |
| --- | --- | --- | --- | --- | --- |
| NC_022584 | I-G2 | Uncertain | | *australis* | |
| NZ_JVSO00000000 | 142_SOLI | *oligofermentans* | | *cristatus* | |
| NZ_JWGG00000000 | 1014_SOLI | *oligofermentans* | | *cristatus* | |
| NZ_JWGF00000000 | 1015_SOLI | *oligofermentans* | | *cristatus* | |
| NZ_JUTP00000000 | 771_SOLI | *oligofermentans* | | *gordonii* | |
| NZ_JYOZ00000000 | I141 | *sanguinis* | | *gordonii* | |
| NZ_JYGN00000000 | UB10712 | *mitis* | | *gordonii* | |
| NZ_ACOI00000000 | 2_1_36FAA | Uncertain | | *gordonii* | |
| NZ_JUSY00000000 | 787_SOLI | *oligofermentans* | | *gordonii* | |
| NZ_JYOV00000000 | UC6950A | *mitis* | | *infantis* | |
| NZ_JYGT00000000 | UC921A | *mitis* | | *infantis* | |
| NZ_ALCH00000000 | SPAR10 | *mitis* | | *infantis* | |
| NZ_JUOS00000000 | 900_SORA | *oralis* | | *infantis* | |
| NZ_JWAJ00000000 | 1172_SPSE | *pseudopneumoniae* | | *infantis* | |
| NZ_JVHE00000000 | 434_SPSE | *pseudopneumoniae* | | *infantis* | |
| NZ_AJML00000000 | SK140 | Uncertain | | *infantis* | |
| NZ_JVFV00000000 | 469_SPSE | *pseudopneumoniae* | | *infantis* | |
| NZ_JVKV00000000 | 342_SPSE | *pseudopneumoniae* | | *infantis* | |
| NZ_AFUO00000000 | bv.2.F0392 | *mitis* | | *oralis ssp. dentisani* | |
| NZ_JYGM00000000 | COL85/1862 | *mitis* | | *oralis ssp. oralis* | |
| NZ_JYGR00000000 | SK141 | *oralis* | | *oralis ssp. oralis* | |
| NZ_JYGO00000000 | OP51 | *mitis* | | *oralis ssp. oralis* | |
| NZ_JYGU00000000 | UC5873 | *mitis* | | *oralis ssp. tigurinus* | |
| NZ_JYPA00000000 | VT517 | *sanguinis* | | *parasanguinis* | |
| NZ_JWCQ00000000 | 1116_SGOR | *gordonii* | | Uncertain | |
| NZ_JWGQ01000123 | 1004_SSPC | Uncertain | | *vestibularis* | |
