# Supplementary material for: Comparative genomics and evolution of the amylase-binding proteins of oral streptococci
Source: BMC Microbiol. 2017 Apr 20;17:94. doi: 10.1186/s12866-017-1005-7 (PMC5399409; doi:10.1186/s12866-017-1005-7)
Supplement: Supplementary file 2 — Amylase-binding protein A-like subgroup comparison. Includes NCBI protein identifiers, molecular weights, and N-terminal sequences. (DOCX 107 kb) [file 12866_2017_1005_MOESM2_ESM.docx]

**Table S2** Amylase-binding protein A-like subgroup comparison

| Subgroup | Organism | Protein identifier | AbpA MW (kDa)  predicted | AbpA calc. MW (kDa) from blot | N-terminal sequence  *in silico* or experimental (bold) |
| --- | --- | --- | --- | --- | --- |
| 1 | S. [multispecies]  *S. gordonii* Challis CH1  *S. gordonii* UB10712^a^  *S. sp.* 2_1_36FAA  *S. gordonii* 787_SOLI^b^  *S. gordonii* KCOM1506 | WP_008810020.1 | 20.5  20.5  20.5  20.5  20.5  20.5 | 20.5  20.5  ND  ND  ND | ADEATDAARNND |
|  |  |  |  |  |  |
| 1 | *S. gordonii*^c^ | AAC38579.1 | 20.5 | 20 | ADEATDAARNND |
| 1 | *S. gordonii* 771SOLI^b^ | WP_048775236.0 | 20.8 | ND | ADEATDAARNND |
| 1 | *S. gordonii* G9B | WP_045504766.1 | 20.5 | 20 | **ADEATDAARKND** |
| 1 | S. [multispecies]  *S. gordonii* IE35  *S. gordonii* I141^d^ | WP_045773634.1 | 20.6  20.6  20.6 | ND  20 | **ADEATDAARKND** |
| 1 | *S. cristatus* CC5A  (ATCC 49999) | WP_045498381.1 | 21.4 | 26 | **ADEATNAARQGD** |
|  |  |  |  |  |  |
| 2 | S. [multispecies]  *S. cristatus* CR3  *S. cristatus* CR311  (ATCC 51100)  *S. cristatus* 142SOLI^b^ | WP_005591359.1 | 22.3  22.3  22.3  22.3 | 28  30  ND | ENPINGGANTPGA  **ENPINGGANT** |
| 2 | *S. cristatus* 1014SOLI^b^  *S. cristatus* 1015SOLI^b^ | WP_048764310.1 | 22.3 | ND | DNSINGGANTPGA |
| 2 | *Gemella haemolysans* | WP_003145915.1 | 21.9 | ND | ENPINGGANKPGA |
| 3 | *S. salivarius* [multispecies]  *S. salivarius* KB005  *S. salivarius* M18  *S. salivarius* CCHSS3  *S. salivarius* 57.1  S. salivarius 726SSAL  S. salivarius 1270SSAL | WP_002886387.1 | 22.8  22.8  22.8  22.8  22.8 | 29  ND  ND  ND | **DINGGANTPGA** |
| 3 | *S. salivarius* UC3162 | WP_045768729.1 | 22.8 | 29 | **DINGGANTPGA** |
| 3 | *S. sp.* HSISS3 | WP_021151344.1 | 22.7 | ND | DINGGANTPGA |
| 3 | *S. oralis* ssp. *oralis* COL85/1862^b^ | WP_045593772.1 | 22.7 | 29 | **DINGGANTPGA** |
| 3 | *S. oralis* ssp. *tigurinus* UC5873^b^ | WP_045616600.1 | 22.7 | 30 | **DINGGANTPGA** |
| 3 | *S. parasanguinis* C1A | WP_031574136.1 | 22.2 | ND | DNNGGANTPRG |
| 3 | *S. parasanguinis* 1287SPAR | WP_049486258.1 | 21.2 | ND | DNNGGANTPRG |
| 3 | *S. parasanguinis* 392SPAR | WP_049523112.1 | 21.7 | ND | DNNGGANTPRG |
|  |  |  |  |  |  |
| 4 | S. [multispecies]  *S. oralis* ssp. *oralis* OP51^b,e^  *S. oralis* ssp. *oralis* SK141^b,e^ | WP_033629133.1 | 22.1 | 25  26 | **SDNSYSSNALI** |
| 4 | *S.* *infantis* UC921A^b^ | WP_045613093.1 | 22.3 | 26 | **SDNSYSSNALI** |
| 4 | *S.* *infantis* UC6950A^b^ | WP_045762490.1 | 24.4 | 30 | **SDNSYSSNALI** |
| 4 | *S. infantis* SPAR10^b^ | WP_004250358.1 | 22.1 | ND | SDNSYSSNALI |
| 4 | *S. parasanguinis* 349SPAR | WP_049490637.1 | 20.9 | ND | SDNSYSSNALI |
| 5 | S. [multispecies]  *S. parasanguinis* FW213  *S. parasanguinis* ATCC 15912  *S. parasanguinis* VT517^f,g^ | WP_013904381 | 22.5 | ND  ND  22 | **QGENPSASNQLI** |
| 5 | *S. parasanguinis* MGH413 | WP_045760080.1 | 22.7 | 21 | **QGENPSSSNQLI** |
|  |  |  |  |  |  |
| 5 | *S. parasanguinis* F0449 | WP_003015345.1 | 24.0 | ND | QGENPNASNQLI |
| 5 | *S. australis* ATCC700641 | WP_006597049.1 | 22.2 | ND | QGENPNASNQLI |
| 5 | *S. sp.* I-G2 | WP_042361305.1 | 22.5 | ND | QGENPNASNQLI |
| 5 | *S. infantis* 342SPSE^b^ | WP_049509170.1 | 22.3 | ND | QGENPNASNQLI |
| 5 | *S. infantis* SK140^b^ | WP_000756778.1 | 22.4 | ND | QGENPASSNQLI |
| 5 | *S. infantis* 1172SPSE^b^ | WP_049549418.1 | 23.6 | ND | QGENPASSNQLI |
| 5 | *S. infantis* 469SPSE^b^ | WP_049530055.1 | 22.2 | ND | QGENPASSNQLI |
| 5 | *S. oralis* ssp. *dentisani* F0392^b^ | WP_000757007.1 | 22.3 | ND | QGENPNSSNQLI |
| 5 | *S. infantis* SK1076 | WP_006150531.1 | 23.1 | ND | QGENPASSNQLI |
| 5 | S. [multispecies]  *S. vestibularis* F0396  *S. vestibularis* ATCC49124  *S. sp.* 1004SSPC | WP_003092326.1 | 22.9 | ND  ND  ND | QGENPKNSNQLT |
|  |  |  |  |  |  |

Blue font, strains tested in this study.

ND = amylase-ligand binding assay not done.

^a^Previously identified as NCTC 10712 in our collection; reclassified as *S. gordonii*, strain UB10712 upon whole genomic sequencing and multilocus sequence analysis.

^b^Strains have been reclassified as indicated above from *S. mitis* (Jensen A, Scholz CFP, and Kilian M. Re-evaluation of the taxonomy of the Mitis group of the genus Streptococcus based on whole genome phylogenetic analysis, and proposed reclassification of *Streptococcus dentisani* as *Streptococcus oralis* subsp. *dentisani* comb. nov., *Streptococcus tigurinus* as *Streptococcus oralis* subsp. *tigurinus* comb. nov., and *Streptococcus oligofermentans* as a later synonym of *Streptococcus cristatus*. Int J Syst Evol Microbiol., 2016 (in press).

^c^Blot done previously, (Rogers JD, Haase EM, Brown AE, Douglas CWI, Gwynn JP, and Scannapieco FA. Identification and analysis of a gene (*abpA*) encoding a major amylase-binding protein in *Streptococcus gordonii*. Microbiology, 144:1223-1233, 1998). Sequence of this Challis is slightly different from database CH1.

^d^Formerly *S. sanguinis* I141, reclassified as *S. gordonii* upon whole genomic sequencing and multilocus sequence analysis.

^e^Genomes of *S. oralis* OP51 and *S. oralis* SK141 are 99.9% identical; considered derivatives (Jensen et al., 2016).

^f^Genomes of *S. parasanguinis* VT517 and *S. parasanguinis* FW213 are 99.97% identical.

^g^Genomes of *S. parasanguinis* VT517 and *S. parasanguinis* ATCC 15912 are 96% identical.
