# Supplementary material for: Comparative genomics and evolution of the amylase-binding proteins of oral streptococci
Source: BMC Microbiol. 2017 Apr 20;17:94. doi: 10.1186/s12866-017-1005-7 (PMC5399409; doi:10.1186/s12866-017-1005-7)
Supplement: Supplementary file 3 — Amylase-binding protein B-like comparison. Includes NCBI protein identifiers, molecular weights, and N-terminal sequences. (DOCX 89 kb) [file 12866_2017_1005_MOESM3_ESM.docx]

**Table S3** Amylase-binding protein B-like comparison

| Organism | Protein identifier | Function | AbpB MW  (kDa)  predicted | AbpB  ca. MW (kDa) from blot | N-terminal sequence |
| --- | --- | --- | --- | --- | --- |
| S. [gordonii]  *S. gordonii* Challis CH1  *S. gordonii* Challis (FAS) | WP_011999704.1 | Peptidase C69 | 72.7  72.7 | 82  82^b^ |  |
| S. [gordonii]  *S. gordonii* G9B  *S. gordonii* IE35 | WP_045505246.1 | Peptidase C69 | 72.7 72.7 | 82^b^  ND |  |
| S. [multispecies]  *S. parasanguinis* FW213  *S. parasanguinis* VT517 | WP_014714017.1 | Peptidase C69  AbpB  Surface-anchored dipeptidase | 71.8  71.8 | ND  84 | DITERVKALNL |
| S. [sanguinis]  *S. gordonii* I141 | WP_045772684.1 | Surface-anchored dipeptidase | 72.8 | 84 | SEDVIFGRQLT |
| *S. cristatus* CC5A | WP_045500372.1 | Peptidase C69  Surface-anchored dipeptidase | 72.9 | 84 | T- TVKSISGLG |
| S. cristatus  *S. cristatus* CR311 (ATCC 51100)  *S. cristatus* CR3 | WP_005591575.1 | Peptidase C69  Peptidase C69  Surface-anchored dipeptidase | 72.9 | 84  82^b^ | Indeterminant |
| S. [gordonii]  *S. gordonii* UB10712^a^ | WP_045634462.1 | Surface-anchored dipeptidase | 72.8 | 82 |  |
| *S. cristatus* 142_SOLI^c^ | WP_048791919.1 | Peptidase C69 | 73.7 | ND |  |
| *S. cristatus* 1015_SOLI^c^ | WP_048766081.1 | Peptidase C69 | 72.5 | ND |  |
| *S. australis* ATCC 700641 | WP_006597304.1 | Peptidase C69 | 72.6 | 86 | SEGDGVGGKPV |
| *S. parasanguinis* C1A | WP_031575668.1 | Peptidase C69 | 72.8 | ND |  |
| *S. parasanguinis* F0449 | WP_003017485.1 | Peptidase C69 | 72.7 | ND |  |
| *S. parasanguinis* MGH413 | WP_045759972.1 | Peptidase C69 | 74.1 | 87^b^ |  |
| *S. gordonii.* 2_1_36FAA | WP_008808186.1 | Peptidase C69 | 72.7 | ND |  |
| *S. australis.* I-G2 | WP_023027108.1 | Peptidase C69 | 65.0 | ND |  |

Blue font, strains tested in this study.

^a^This organism has been recently reclassified as *S. gordonii*, unspecified strain.

^b^Brown AE, Rogers JD, Haase EM, and Scannapieco FA. Prevalence of the amylase-binding proteins A gene (*abpA*) in oral streptococci. J Clin Microbiol 37:4081-4085, 1999.

^c^Strains have been reclassified as indicated above based on whole genome core phylogeny. From *S. mitis* (Jensen A, Scholz CFP, and Kilian M. Re-evaluation of the taxonomy of the Mitis group of the genus Streptococcus based on whole genome phylogenetic analysis, and proposed reclassification of *Streptococcus dentisani* as *Streptococcus oralis* subsp. *dentisani* comb. nov., *Streptococcus tigurinus* as *Streptococcus oralis* subsp. *tigurinus* comb. nov., and *Streptococcus oligofermentans* as a later synonym of *Streptococcus cristatus*. Int J Syst Evol Microbiol., 2016 (in press).

ND, not determined.
