# Supplementary material for: Comparative genomics and evolution of the amylase-binding proteins of oral streptococci
Source: BMC Microbiol. 2017 Apr 20;17:94. doi: 10.1186/s12866-017-1005-7 (PMC5399409; doi:10.1186/s12866-017-1005-7)
Supplement: Supplementary file 4 — CLUSTAL alignment of AbpB-like protein sequences. (DOCX 167 kb) [file 12866_2017_1005_MOESM4_ESM.docx]

**Fig. S1** CLUSTAL alignment of AbpB-like protein sequences

S.parasanguinis_FW213 MKKWLLKLSLVVMTLLLLPIQAVQACCGFIIGRQLTKDGTTLFGRTEDYPYYPNGGKHNK

S.parasanguinis_VT517^a^ ^b^ MKKWLLKLSLVVMTLLLLPIQAVQACCGFIIGRQLTKDGTTLFGRTEDYPYYPNGGKHNK

S.parasanguinis_MGH413^a^ ^b^ MKKWLLKLSLVAMTLLLLPIQAVQACCGFIIGRQLTKDGTTLFGRTEDYPYYPNGGKHNK

S.cristatus_CC5A^a^ ^b^ MKKTIFKIAISLMAIMLLPIQAVQACCGFIIGRHLTKDGSTLFGRTEDYPYYPNGGKHNK

S.cristatus_CR3^a^ ^b^ MKKTIFKIAISLMAIMLLPIQAVQACCGFIIGRHLTKDGSTLFGRTEDYPYYPNGGKHNK

S.cristatus_CR311/ATCC51100^c^ MKKTIFKIAISLMAIMLLPIQAVQACCGFIIGRHLTKDGSTLFGRTEDYPYYPNGGKHNK

S.gordonii_CH1^d^ MKKLTFKVVIAVMAILMLPIQAVQACCGFIFGRQLTADGSTMFGRTEDYPYYPNGGKHNK

S.gordonii_G9B^a^ MKKLTFKVVIAVMAILMLPIQAVQACCGFIFGRQLTADGSTMFGRTEDYPYYPNGGKHNK

S.gordonii_IE35 MKKLTFKVVIAVMAILMLPIQAVQACCGFIFGRQLTADGSTMFGRTEDYPYYPNGGKHNK

S.gordonii_I141^a^ ^b^ MKKLTFKVVIAVMAILMLPIQAVQACCGFIFGRQLTADGSTMFGRTEDYPYYPNGGKHNK

*** :*: : *::::*************:**:** **:*:******************

S.parasanguinis_FW213 NFVVVDAKNYKEGDQLEDESNGFTYPHAASEMKYTATYDSARGDGSNGAFGEHGFNEAGV

S.parasanguinis_VT517 NFVVVDAKNYKEGDQLEDESNGFTYPHAASEMKYTATYDSARGDGSNGAFGEHGFNEAGV

S.parasanguinis_MGH413 NFVVVDAKNYKEGDQLEDESNGFTYPHAASEMKYTATYDSARGDGSNGAFGEHGFNEAGV

S.cristatus_CC5A NYVVVEAKKYKEGDQIVDESNGFTYPRASSEMKYTATYDSARGDGSNGAFGEHGFNEAGV

S.cristatus_CR3 NYVVVEAKKYKEGDQIVDESNGFTYPRAASEMKYTATYDSARGDGSNGAFGEHGFNEAGV

S.cristatus_CR311/ATCC51100 NYVVVEAKKYKEGDQIVDESNGFTYPRAASEMKYTATYDSARGDGSNGAFGEHGFNEAGV

S.gordonii_CH1 NYVVVEGKNYKEGDQIVDESNGFTYPHAANEMKYTATYDSARGDGSNGAFGEHGFNEAGV

S.gordonii_G9B NYVVVEGKNYKEGDQIVDESNGFTYPHAANEMKYTATYDSARGDGSNGAFGEHGFNEAGV

S.gordonii_IE35 NYVVVEGKNYKEGDQIVDESNGFTYPHAANEMKYTATYDSARGDGSNGAFGEHGFNEAGV

S.gordonii_I141 NYVVVEGKNYKEGDQIVDESNGFTYPHAANEMKYTATYDSARGDGSNGAFGEHGFNEAGV

*:***:.*:******: *********:*:.******************************

S.parasanguinis_FW213 SMTSTVTAIPNKKVLKTDPLTENGIPEAAMLDVVLPRVKSAREGVEFLAKVIEEKGSAEG

S.parasanguinis_VT517 SMTSTVTAIPNKKVLKTDPLTENGIPEAAMLDVVLPRVKSAREGVEFLAKVIEEKGSAEG

S.parasanguinis_MGH413 SMTSTVTAIPNKKVLKTDPLTEKGIPEAAMLDVVLPRVKSAREGIELLAKVIEEKGSAEG

S.cristatus_CC5A SMSSTVTAIPNKKVLEKDPLKADGLAEAAMLDVILPRAKTAREAIELLGKVIQEKGSAEG

S.cristatus_CR3 SMSSTVTAIPNKKVLEKDPLKADGLAEAAMLDVVLPRAKTAREAIELLGKVIQEKGSAEG

S.cristatus_CR311/ATCC51100 SMSSTVTAIPNKKVLEKDPLKADGLAEAAMLDVVLPRAKTAREAIELLGKVIQEKGSAEG

S.gordonii_CH1 SMTSTVTAIPNKKVLAKDPLKADGLPEAAMLDVILPRAKSAREAIELLAKVIEEKGSAEG

S.gordonii_G9B SMTSTVTAIPNKKVLAKDPLKADGLPEAAMLDVILPRAKSAREAIELLAKVIEEKGSAEG

S.gordonii_IE35 SMTSTVTAIPNKKVLAKDPLKADGLPEAAMLDVILPRAKSAREAIELLAKVIEEKGSAEG

S.gordonii_I141 SMTSTVTAIPNKKVLAKDPLKADGLPEAAMLDVILPRAKSAREAIELLAKVIEEKGSAEG

**:************ .***. .*: *******:***.*:***.:*:*.***:*******

S.parasanguinis_FW213 NVVVFADQKETWYMEILSGHQYVAVKVPEDKYAVFANTYYLGHVDLNDKENVIASKDVEK

S.parasanguinis_VT517 NVVVFADQKETWYMEILSGHQYVAVKVPEDKYAVFANTYYLGHVDLNDKENVIASKDVEK

S.parasanguinis_MGH413 NTVVIADQKETWYMEILSGHQYVAVKVPEDKYAVFANTYYLGHVNLNDTENVIASKDVEK

S.cristatus_CC5A NTVVVADQKETWYMEILSGHQYVAVKVPEDKYAVFANTYYLGHVDLKDKENVIASEDVEK

S.cristatus_CR3 NTVVVADQKETWYMEILSGHQYVAVKVPEDKYAVFANTYYLGHVDLKDKENVIASEDVEK

S.cristatus_CR311/ATCC51100 NTVVVADQKETWYMEILSGHQYVAVKVPEDKYAVFANTYYLGHVDLKDKENVIASEDVEK

S.gordonii_CH1 NTVVVADQKETWYMEILSGHQYVAVKVPEDKYAVFANTYYLGHVDLNDKENVIASKDVEK

S.gordonii_G9B NTVVVADQKETWYMEILSGHQYVAVKVPEDKYAVFANTYYLGHVDLNDKENVIASKDVEK

S.gordonii_IE35 NTVVVADQKETWYMEILSGHQYVAVKVPEDKYAVFANTYYLGHVDLNDKENVIASKDVEK

S.gordonii_I141 NTVVVADQKETWYMEILSGHQYVAVKVPEDKYAVFANTYYLGHVDLNDKENVIASKDVEK

*.**.***************************************:*:*.******:****

S.parasanguinis_FW213 VAKESGNYKTDKDGNFHIAKSYGPDKYAEGDRSRTYAGITLLDPKSKVTYEDDEYELFRS

S.parasanguinis_VT517 VAKESGNYKTDKDGNFHIAKSYGPDKYAEGDRSRTYAGITLLDPKSKVTYEDDEYELFRS

S.parasanguinis_MGH413 VAKESGSYKTDKDGNFHIAKSYGPEKYAEGDRSRTYAGITLLDPKSKVTYEDDEYELFRS

S.cristatus_CC5A VAKEAGNYKTDKDGNFHIAKSYGPEKYAEGDRSRTYAGITLLDPKSKITYEDEEYELFRS

S.cristatus_CR3 VAKEAGNYKTDKDGNFHIAKSYGPEKYAEGDRSRTYAGITLLDPKSKITYEDEEYELFRS

S.cristatus_CR311/ATCC51100 VAKEAGNYKTDKDGNFHIAKSYGPEKYAEGDRSRTYAGITLLDPKSKITYEDEEYELFRS

S.gordonii_CH1 VAQEAGNYKTDKDANFHIAKSYGPEKYAEGDRSRTYAGITLLDPKSKITYEDDEYELFRS

S.gordonii_G9B VAQEAGNYKTDKDGNFHIAKSYGPEKYAEGDRSRTYAGITLLDPKSKITYEDDEYELFRS

S.gordonii_IE35 VAQEAGNYKTDKDGNFHIAKSYGPEKYAEGDRSRTYAGITLLDPKSKITYEDDEYELFRS

S.gordonii_I141 VAQEAGNYKTDKDGNFHIAKSYGPEKYAEGDRSRTYAGITLLDPKSKITYEDDEYELFRS

**:*:*.******.**********:**********************:****:*******

S.parasanguinis_FW213 PTDPNKKFTLEDAFAFQRNRFEHLNGRFVPDDQIGVKKQGDNGSNDTVRKDQYKYALGNE

S.parasanguinis_VT517 PTDPNKKFTLEDAFAFQRNRFEHLNGRFVPDDQIGVKKQGDNGSNDTVRKDQYKYALGNE

S.parasanguinis_MGH413 PTDPNKKFTLEDAFALQRNRFEHLNGRFVPDDQIGVKKQGDNGSNDAVRKDQYKYALGNE

S.cristatus_CC5A PTDPNKKFTLEDAFALQRNRFEHLNGRFVPDDQIGVKKQGDDGANDAVRKDQYKYALGNE

S.cristatus_CR3 PTDPNKKFTLEDAFALQRNRFEHLNGRFVPDDQIGVKKQGDDGANDAVRKDQYKYALGNE

S.cristatus_CR311/ATCC51100 PTDPNKKFTLEDAFALQRNRFEHLNGRFVPDDQIGVKKQGDDGANDAVRKDQYKYALGNE

S.gordonii_CH1 PTDPNKKFTLEDAFALQRNRFEHLNGRFIPDDQIGVKKQGDNGANDAVRKDQYKYALGNE

S.gordonii_G9B PTDPNKKFTLEDAFALQRNRFEHLNGRFVPDDQIGVKKQGDNGANDAVRKDQYKYALGNE

S.gordonii_IE35 PTDPNKKFTLEDAFALQRNRFEHLNGRFVPDDQIGVKKQGDNGANDAVRKDQYKYALGNE

S.gordonii_I141 PTDPNKKFTLEDAFALQRNRFEHLNGRFIPDDQIGVKKQGDNGANDAVRKDQYKYALGNE

***************:************:************:*:**:*************

S.parasanguinis_FW213 NVIDAHVYQINPNLPKSFGGTLWLGMGPSRNTPYVPFYGNVKDTYKAFKPQTATYDPNSW

S.parasanguinis_VT517 NVIDAHVYQINPNLPKSFGGTLWLGMGPSRNTPYVPFYGNVKDTYKAFKPQTATYDPNSW

S.parasanguinis_MGH413 NVIDAHVYQINPNLPKSFGGTVWLGMGPSRNTPYVPFYGNLKDTYEAFKPQTATYDPNSW

S.cristatus_CC5A NVIDAHVYQIDPKLPKSFGGKVWLGLGPSRNTPYVPFYGNVQDTYKAFKPQTATYDPNSW

S.cristatus_CR3 NVIDAHVYQIDPKLPKSFGGKVWLGLGPSRNTPYVPFYGNVQDTYKAFKPQTATYDPNSW

S.cristatus_CR311/ATCC51100 NVIDAHVYQIDPKLPKSFGGKVWLGLGPSRNTPYVPFYGNVQDTYKAFKPQTATYDPNSW

S.gordonii_CH1 NVIDAHVYQIDPKLPNSFGGKVWLGLGPSRNTPYVPFYGNVQDTYHAFKPQTATYDPNSW

S.gordonii_G9B NVIDAHVYQIDPKLPKSFGGKVWLGLGPSRNTPYVPFYGNVQDTYQAFKPQTATYDPNSW

S.gordonii_IE35 NVIDAHVYQIDPKLPKSFGGKVWLGLGPSRNTPYVPFYGNVQDTYQAFKPQTATYDPNSW

S.gordonii_I141 NVIDAHVYQIDPKLPKSFGGKVWLGLGPSRNTPYVPFYGNVQDTYQAFKPQTATYDPNSW

**********:*:**:****.:***:**************::***.**************

S.parasanguinis_FW213 YWTVWHIDNMAINNQDIFGKTVQDHWKALEKQLIIEQEASDAKYKALKDNPEAAKAVEDE

S.parasanguinis_VT517 YWTVWHIDNMAINNQDIFGKTVQDHWKALEKQLIIEQEASDAKYKALKDNPEAAKAVEDE

S.parasanguinis_MGH413 YWTVWHIDNMAINNQDVFGKTVQDHWKALEKQLIIEQEASDAKYKALKDNPEAAKAVEDE

S.cristatus_CC5A YWTVWHIDQMAIKNQDIFGKSVQDHWKMMEKQFIADQEKKDIENYSLKDQPDAAKAAEGK

S.cristatus_CR3 YWTVWHIDQMAIKNQDIFGKSVQDHWKMMEKQFIADQEKKDIENYSLKDQPDAAKAAEGK

S.cristatus_CR311/ATCC51100 YWTVWHIDQMAIKNQDIFGKSVQDHWKMMEKQFIADQEKKDIENYSLKDQPDAAKAAEGK

S.gordonii_CH1 YWTVWHIDQMAIKNQDIFGKTVQDHWKMLEKQFIIDQEKKDIEYYSLKDQPEAAKAAEHK

S.gordonii_G9B YWTVWHIDQMAIKNQDIFGKTVQDHWKMLEKQFIIDQEKKDIEYYSLKDQPEAAKAAEHK

S.gordonii_IE35 YWTVWHIDQMAIKNQDIFGKTVQDHWKMLEKQFIIDQEKKDIEYYSLKDQPEAAKAAEHK

S.gordonii_I141 YWTVWHIDQMAIKNQDIFGKTVQDHWKMLEKQFIIDQEKKDIEYYSLKDQPEAAKAAEHK

********:***:***:***:****** :***:* :** .* : :***:*:****.* :

S.parasanguinis_FW213 VTANALALSKKMFEHFKSYEADMHARLVEAGRKDDPYRASLPDNYKDPEN--PVTPEKPD

S.parasanguinis_VT517 VTANALALSKKMFEHFKSYEADMHARLVEAGRKDDPYRASLPDNYKDPEN--PVTPEKPD

S.parasanguinis_MGH413 VTANALALSKKLFEHFKSYEADMHAHLISLGRKDDPYRASKPDDYKDPEPEKPVQPEKPV

S.cristatus_CC5A VTKDSLEMSDRLFQHFKDYEKMMEKRLEAAGRKSDPYRASQPDDKQDPS-----TPEKPD

S.cristatus_CR3 VTKDSLEMSDRLFQHFKDYEKMMEKRLEAAGRKSDPYRASQPDDKQDPS-----TPEKPD

S.cristatus_CR311/ATCC51100 VTKDSLEMSDRLFQHFKDYEKMMEKRLEAAGRKSDPYRASQPDDKQDPS-----TPEKPD

S.gordonii_CH1 VTKDALDLSDRLFQHFKDYEKLMEKQLEAAGRKSDPYRASQPDDKQDPS-----TPEKPD

S.gordonii_G9B VTKDALDLSDRLFQHFKDYEKLMEKQLEAAGRKSDPYRASQPDDKQDPS-----TPEKPD

S.gordonii_IE35 VTKDALDLSDRLFQHFKDYEKLMEKQLEAAGRKSDPYRASQPDDKQDPS-----TPEKPD

S.gordonii_I141 VTKDALDLSDRLFQHFKDYEKLMEKQLEAAGRKSDPYRASQPDDKQDPS-----TPEKPD

** ::* :*.::*:***.** *. :* ***.****** **: :**. ****

S.parasanguinis_FW213 PGTKPE-----------VNPNN-----PVVEPKKPLPIQTKVNAEGNPNNRFGYAATKDK

S.parasanguinis_VT517 PGTKPE-----------VNPNN-----PVVEPKKPLPIQTKVNAEGNPNNRFGYAATKDK

S.parasanguinis_MGH413 EPEKPVQPEKPVEPEKPVQPEKPVQPEKPVQPEKTQPIQTKVNEEGNPNNRFGYAATKDK

S.cristatus_CC5A TPKPPQKPQITDDANKTIIPAPDYT-------AAPPTHDIPANAEGNPNNRFGYAAKANV

S.cristatus_CR3 TPKPPQKPQITDDANKTIIPAPDYT-------AAPPTHDIPANAEGNPNNRFGYAAKANV

S.cristatus_CR311/ATCC51100 TPKPPQKPQITDDANKTIIPAPDYT-------AAPPTHDIPANAEGNPNNRFGYAAKANV

S.gordonii_CH1 TPKTPEKPKATQEVNKNALPAEYYV-------AAPPTHDIPANAEGNPNNRFGYAATANV

S.gordonii_G9B TPKTPEKPKATEEVNKNVLPAEYYV-------AAPPTHDIPANAEGNPNNRFGYAATANV

S.gordonii_IE35 TPKTPEKPKATEEVNKNVLPAEYYV-------AAPPTHDIPANAEGNPNNRFGYAATANV

S.gordonii_I141 TPKTPEKPKATEEVNKNVLPAEYYV-------AAPPTHDIPANAEGNPNNRFGYAATANV

* * : .* ************. :

S.parasanguinis_FW213 ESYVSTRVETFVK--PEEKVQDPSE---AVSAGNGGNENNRFGSSYDVAS------AGTF

S.parasanguinis_VT517 ESYVSTRVETFVK--PEEKVQDPSE---AVSAGNGGNENNRFGSSYDVAS------AGTF

S.parasanguinis_MGH413 ESYVSTKVETFVK--PEAKAQDPSE---AVSAGNGGNENNRFGSSFAKAT------VETF

S.cristatus_CC5A QVFGQPSVRTEVPQVQNQTPQAPSQAPQITVANAEGNPNNRFGYAANSDNGKNFKNVETF

S.cristatus_CR3 QVFGQPSVRTEVPQVQNQTPQAPSQAPQITVANAEGNPNNRFGYTANSDNGKNFKNVETF

S.cristatus_CR311/ATCC51100 QVFGQPSVRTEVPQVQNQTPQAPSQAPQITVANAEGNPNNRFGYTANSDNGKNFKNVETF

S.gordonii_CH1 QVFGQTSTRAEAPQVQSQASQEPSQAPQTTVANAEGNPNNRFGFAGNYEGGKDFKNIGTF

S.gordonii_G9B QVFGQTSTRAEAPQVQSQASQEPSQAPQTTVANAEGNPNNRFGFAGNYEGGKDFKNIGTF

S.gordonii_IE35 QVFGQTSTRAEAPQVQSQASQEPSQAPQTTVANAEGNPNNRFGFAGNYEGGKDFKNIGTF

S.gordonii_I141 QVFGQTSTRAEAPQVQSQAFQEPSQAPQTTVANAEGNPNNRFGFAGNYEGGKDFKNIGTF

: : . ..: . . * **: . * ** ***** : **

S.parasanguinis_FW213 QNTNYSYNVHP

S.parasanguinis_VT517 QNTNYSYNVHP

S.parasanguinis_MGH413 PNTNYSYNVHP

S.cristatus_CC5A VNTK-------

S.cristatus_CR3 VNTK-------

S.cristatus_CR311/ATCC51100 VNTK-------

S.gordonii_CH1 SNGN-------

S.gordonii_G9B SNGN-------

S.gordonii_IE35 SNGN-------

S.gordonii_I141 SNGN-------

* :

^a^AbpB sequences from primer walking with degenerate primers to known AbpB sequences.

^b^Streptococcal strains sequenced in this study.

^c^Alternative name, *Streptococcus cristatus* ATCC 51100.

^d^AbpB demonstrated to be a dipeptidase (**Chaudhuri B, Paju S, Haase EM, Vickerman MM, Tanzer JM, Scannapieco FA.** 2008. Amylase-binding protein B of *Streptococcus gordonii* is an extracellular dipeptidyl-peptidase. Infect Immun **76:**4530-4537.

Blue font: signal sequence predicted by PROSITE.

Red font: residues are part of N-terminal sequence determined experimentally.

Aqua highlight: similar N-terminal sequences.
