# Supplementary material for: Comparative genomics and evolution of the amylase-binding proteins of oral streptococci
Source: BMC Microbiol. 2017 Apr 20;17:94. doi: 10.1186/s12866-017-1005-7 (PMC5399409; doi:10.1186/s12866-017-1005-7)
Supplement: Supplementary file 5 — Novel amylase-binding protein comparison. Includes NCBI protein identifiers, molecular weights, and N-terminal sequences. (DOCX 82 kb) [file 12866_2017_1005_MOESM5_ESM.docx]

**Table S4** Novel amylase-binding protein comparison

| Organism | Protein identifier | Function | MW (kDa) predicted | MW (dDa) from blot | N-terminal sequence |
| --- | --- | --- | --- | --- | --- |
|  |  | **LysM superfamily**  **Peptidoglycan-binding protein** |  |  |  |
| S. [*cristatus*]  *S. cristatus* CR3    *S. cristatus* CR311/  ATCC 51100  *S. cristatus* 142_SOLI^a^ | WP_005591387.1 | Peptidoglycan-binding  LysM domain-containing protein  Transglycosylase-like domain protein  Peptidoglycan-binding protein | 22.5  22.5  22.5 | 28  ND  ND | ESYTVKGDTL |
| S. [multispecies]  *S. cristatus* 1015_SOLI^a^  *S. cristatus* 1014_SOLI^a^  *S. cristatus* CC5A | WP_045499911.1 | Peptidoglycan-binding protein  Peptidoglycan-binding protein  Peptidoglycan-binding protein  LysM domain-containing protein | 22.5  22.5  22.5 | ND  ND  26 | ASYTVKA(A)RQL |
| *S. cristatus* AS 1.3089^a^ | WP_015606085.1 | Hypothetical protein  Hypothetical 1872-10645 | 22.3 | ND |  |
| *S. sp.* 1116_SGOR^a^ | WP_048770108.1 | Peptidoglycan-binding protein | 21.8 | ND |  |
|  |  | **Periplasmic-binding domain Type 2 superfamily**  **Domain: PRK11917** |  |  |  |
| *S. mitis* [multispecies]  *S. oralis* ssp. *oralis* SK141^a^  *S. oralis* ssp. *oralis* OP51^a^ | WP_033630283.1 | Glutamine ABC transporter substrate-binding protein | 28.2 | 26 | DTSVADIQKRG |
| S. oralis ATCC 49296 | WP_000726187.1 | Glutamine ABC transporter substrate-binding protein | 29.4 | ND |  |
| *S. oralis* ssp. *tigurinus*  UC5873^a^ | WP_045617532.1 | Glutamine ABC transporter substrate-binding protein | 29.4 | ND |  |
| *S.* *oralis* ssp. *oralis* COL85/1862^a^ | WP_045590578.1 | Glutamine ABC transporter substrate-binding protein | 29.4 | ND |  |
| *S. mitis* B6 | WP_000726181.1 | ABC type amino acid transporter, substrate binding | 29.5 | ND |  |
| *S. pneumoniae* R6 | WP_000726170.1 | ABC type amino acid transporter, substrate binding | 29.5 | ND |  |
|  |  | **Hypothetical protein** |  |  |  |
|  |  |  |  |  |  |
| *S. infantis* UC921A^a^ | WP_045613961.1 | Hypothetical protein  IgA Fc receptor | 25.9 | 30 | ATEVPSIPGVT |
| *S. infantis* SPAR_10^a^ | WP_004252463.1 | Hypothetical protein | 23.9 | ND |  |
| S. [multispecies]  *S. oralis* ssp. *oralis* SK141^a^  *S. oralis* ssp. *oralis* OP51^a^ | WP_033629412.1 | Hypothetical protein  Hypothetical protein | 24.4  24.4 | 30  30 | ATEVPSIPGVT  ATEVPSIPGVT |
| *S. infantis* SK1076 | WP_006150576.1 | Hypothetical protein HMPREF_1426 | 23.9 | ND |  |
|  |  | **Cell wall binding domain**  **Glucan binding domain**  **Choline-binding protein A** |  |  |  |
| *S. mitis* SK145 | WP_045606492.1 | Choline binding protein A | 37.6 | 37 | DTNNGYSESGV |
| *S. mitis* SK137 | WP_045597421.1 | Choline binding protein A | 37.3 | ND |  |
| *S. mitis* 29/42 | WP_020903284.1 | Hypothetical protein M060_6295 | 37.1 | ND |  |
| *S. mitis* SK637 | WP_033687884.1 | Choline binding protein A | 38.1 | ND |  |
| *S. pneumoniae* SMRU2014 | [WP_050243109.1](https://www.ncbi.nlm.nih.gov/protein/WP_050243109.1) | Choline binding protein Cbp1 | 39.4 | ND |  |
| *S. mitis* SK271 | WP_033685289.1 | Choline binding protein A | 32.9 | ND |  |
| *S. mitis* NS51^b^ | ABS18283.1 | Amylase-binding protein C | 32.3 | 36 | DSQAQYSNGV |

Blue font, strains tested in this study.

^a^Strains have been reclassified as indicated above based on whole genome core phylogeny. From *S. mitis* (Jensen A, Scholz CFP, and Kilian M. Re-evaluation of the taxonomy of the Mitis group of the genus Streptococcus based on whole genome phylogenetic analysis, and proposed reclassification of *Streptococcus dentisani* as *Streptococcus oralis* subsp. *dentisani* comb. nov., *Streptococcus tigurinus* as *Streptococcus oralis* subsp. *tigurinus* comb. nov., and *Streptococcus oligofermentans* as a later synonym of *Streptococcus cristatus*. Int J Syst Evol Microbiol., 2016 (in press).

^b^Vorrasi J, Chaudhuri B, Haase EM, and Scannapieco FA. Identification of amylase binding protein C (AbpC) from *Streptococcus mitis* NS51. Mol Oral Microbiol 25:150-156, 2010.

Sorensen UBS. PLoS One. E: 1-10, 2008. E2683.
