# Supplementary material for: Comparative genomics and evolution of the amylase-binding proteins of oral streptococci
Source: BMC Microbiol. 2017 Apr 20;17:94. doi: 10.1186/s12866-017-1005-7 (PMC5399409; doi:10.1186/s12866-017-1005-7)
Supplement: Supplementary file 6 — CLUSTAL alignment of novel amylase-binding proteins. (DOCX 145 kb) [file 12866_2017_1005_MOESM6_ESM.docx]

**Fig. S2** CLUSTAL alignment of novel amylase-binding proteins

**LysM superfamily peptidoglycan-binding protein**

AbpN_S.cristatus_CC5A_WP_045499911.1 MKVKKLTLAGTIATATLFASTIVASAESYTVKAGDTLSEIAESKKTTVERLVELNKISNP

AbpN_S.cristatus_CR3_WP_005591387.1 MKVKKLTLAGTIATATLFASTIVASAESYTVKAGDTLSEIAESKKTTVERLVELNKISNP

************************************************************

AbpN_S.cristatus_CC5A_WP_045499911.1 DFIMTGQVLELGDLKDVAKQTTSAPAVSPATPAVPAPVTPAAPVATETPVTTETPAVAAT

AbpN_S.cristatus_CR3_WP_005591387.1 DFIMTGQVLELGDLKDVAKQTTSAPAVSPATPAVPAPVTPAAPVATETPVTTETPAVTAT

*********************************************************:**

AbpN_S.cristatus_CC5A_WP_045499911.1 SDYSAYSSDVVLANGNTPGAVGSYAAARMAEMTGVSASTWENIIARESNGQVDAYNPSGA

AbpN_S.cristatus_CR3_WP_005591387.1 SDYSAYSSDVVLANGNTPGAVGSYAAARMAEMTGVSASTWENIIARESNGQVDAYNPSGA

************************************************************

AbpN_S.cristatus_CC5A_WP_045499911.1 SGLFQTMPGWGSTATVEDQIQAAYRAYSAQGLSAWAY

AbpN_S.cristatus_CR3_WP_005591387.1 SGLFQTMPGWGSTATVEDQIQAAYRAYSAQGLSAWAY

*************************************

**Periplasmic-binding domain type 2 superfamily**

AbpN_S.oralis ssp. oralis_COL85/1862_WP_045590578.1 MKKKLFLSALFISLFCLAVAKPVQADTSVADIQKRGELVVGVKQDVPNFGYKDPKTGTYS

AbpN_S.oralis ssp. tigurinus_UC5873_WP_045617532.1 MKKKLFLSALLISLFSLAAAKPVQADTSVADIQKRGKLVVGVKQDVPNFGYKDPKTGTYS

AbpN_S.oralis ssp. oralis_SK141_WP_033630283.1 ----------MISLFGLTAAKPVQADTSVADIQKRGELVVGVKQDVPNFGYKDPKTGTYS

AbpN_S.oralis ssp. oralis_OP51_WP_033630283.1 ----------MISLFGLTAAKPVQADTSVADIQKRGELVVGVKQDVPNFGYKDPKTGTYS

:**** *:.*****************:***********************

AbpN_S.oralis ssp. oralis_COL85/1862_WP_045590578.1 GIETDLAKMIADELKVKIRYVPVTAQTRGPLLDNEQVDMDIATFTITDERKKLYNFTSPY

AbpN_S.oralis ssp. tigurinus_UC5873_WP_045617532.1 GIETDLAKMIADELKVKVRYVPVTAQTRGPLLDNEQVDMDIATFTITDERKKLYNFTSPY

AbpN_S.oralis ssp. oralis_SK141_WP_033630283.1 GIETDLAKMIADELKVKIRYVPVTAQTRGPLLDNEQVDMDIATFTITDERKKLYNFTSPY

AbpN_S.oralis ssp. oralis_OP51_WP_033630283.1 GIETDLAKMIADELKVKIRYVPVTAQTRGPLLDNEQVDMDIATFTITDERKKLYNFTSPY

*****************:******************************************

AbpN_S.oralis ssp. oralis_COL85/1862_WP_045590578.1 YTDASGFLVNKSANIKSIEDLNGKTIGVAQGSITQRLITELGKKKGLTFKFVELGSYPEL

AbpN_S.oralis ssp. tigurinus_UC5873_WP_045617532.1 YTDASGFLVNKSANIKSIEDLNGKTIGVAQGSITQRLITELGKKKGLTFKFVELGSYPEL

AbpN_S.oralis ssp. oralis_SK141_WP_033630283.1 YTDASGFLVNKSANIKSIEDLNGKTIGVAQGSITQRLITELGKKKGLTFKFVELGSYPEL

AbpN_S.oralis ssp. oralis_OP51_WP_033630283.1 YTDASGFLVNKSANIKSIEDLNGKTIGVAQGSITQRLITELGKKKGLTFKFVELGSYPEL

************************************************************

AbpN_S.oralis ssp. oralis_COL85/1862_WP_045590578.1 ITSLHAHRIDAFSVDRSILSGYISKRTELLDDSLKPSDYGIVTKKSNTELSDYLDTLVTK

AbpN_S.oralis ssp. tigurinus_UC5873_WP_045617532.1 ITSLHAHRIDAFSVDRSILSGYISKRTELLDDSFKPSDYGIVTKKSNTELNDYLDTLVTK

AbpN_S.oralis ssp. oralis_SK141_WP_033630283.1 ITSLHAHRIDAFSVDRSILSGYISKRTELLDDSFKPSDYGIVTKKSNTELNDYLDTLVTK

AbpN_S.oralis ssp. oralis_OP51_WP_033630283.1 ITSLHAHRIDAFSVDRSILSGYISKRTELLDDSFKPSDYGIVTKKSNTELNDYLDTLVTK

*********************************:****************.*********

AbpN_S.oralis ssp. oralis_COL85/1862_WP_045590578.1 WSKDGSLQKLYDRYKLKPSSHTAD

AbpN_S.oralis ssp. tigurinus_UC5873_WP_045617532.1 WSKDGSLQKLYDRYKLKPSSHTAD

AbpN_S.oralis ssp. oralis_SK141_WP_033630283.1 WSKDGSLQKLYGRYKLKPSSHTAD

AbpN_S.oralis ssp. oralis_OP51_WP_033630283.1 WSKDGSLQKLYGRYKLKPSSHTAD

*********** ************

**Hypothetical protein**

AbpN_S.infantis_UC921A_WP_045613961.1 MKKVLLTSVLGLSAIASVSYAATEVPSIPGVTASTAGTVTVANAKNNHNNRFGYAISPLR

AbpN_S.oralis ssp. oralis_SK141_WP_033629412.1 MKKVLLTSALALSAIASVSYAATEVPSIPGVTAPTTGTVTEANAKNNHNNRFGYAVSKQ-

AbpN_S.oralis ssp. oralis_OP51_KJQ66935.1 MKKVLLTSALALSAIASVSYAATEVPSIPGVTAPTTGTVTEANAKNNHNNRFGYAVSKQ-

********.*.********************** *:**** **************:*

AbpN_S.infantis_UC921A_WP_045613961.1 KEKAINFAGAKTYPAKPYPGTDGKPLNDSTREFGHNTRFGAGFNATKSTTPATPATPATP

AbpN_S.oralis ssp. oralis_SK141_WP_033629412.1 YDKAYSIAGAITYPAKPYPGTDGKPLNDSTREFGHNTRFGAGFNATKSTTPATPAKPATP

AbpN_S.oralis ssp. oralis_OP51_KJQ66935.1 YDKAYSIAGAITYPAKPYPGTDGKPLNDSTREFGHNTRFGAGFNATKSTTPATPAKPATP

:** .:*** ********************************************.****

AbpN_S.infantis_UC921A_WP_045613961.1 AKPATPATPAKPATPAKPATPAKPATPATPAKPATPAKPAKPATPATPAKPDPKAIPGVT

AbpN_S.oralis ssp. oralis_SK141_WP_033629412.1 AKPATPATPAKPATPATP---------------ATPATPATPAKPATPAKPDPKAIPGVT

AbpN_S.oralis ssp. oralis_OP51_KJQ66935.1 AKPATPATPAKPATPATP---------------ATPATPATPAKPATPAKPDPKAIPGVT

****************.* ****.**.**.****************

AbpN_S.infantis_UC921A_WP_045613961.1 RPTTEIVTEANAANNHNNRFGYAVSPQYKDAYSVSGAETYNTSAPIEGTDGKPLNDSTRE

AbpN_S.oralis ssp. oralis_SK141_WP_033629412.1 RPTTEIVTEANAANNHNNRFGYAVSPQYKDAYSVSGAETYT-SAPIEGTDGKPLNDATRE

AbpN_S.oralis ssp. oralis_OP51_KJQ66935.1 RPTTEIVTEANAANNHNNRFGYAVSPQYKDAYSVSGAETYT-SAPIEGTDGKPLNDATRE

****************************************. **************:***

AbpN_S.infantis_UC921A_WP_045613961.1 LGDNTRFGAGFSK

AbpN_S.oralis ssp. oralis_SK141_WP_033629412.1 FDNNTRFGAGFNK

AbpN_S.oralis ssp. oralis_OP51_KJQ66935.1 FDNNTRFGAGFNK

: :********.*

**Choline-binding protein**

AbpC_S.mitis_NS51_ABS18283.1 MKKVLLTSAVALAAFGAVQAVSADSQAQYS--------------------------NGVV

AbpN_S.mitis_SK137_WP_045597421.1 MKKVLLTSAVALAAFGAVQAVSADTNNGYTESGRVNPKTGKLDVAKYTENEVKSAHTGVV

AbpN_S.mitis_SK145_WP_045606492.1 MKKVLLTSAVALAAFGAVQAVSADTNNGYSESGVVNPKTGRIDVKQYQGNTVVSAHTGVV

************************:: *: .***

AbpC_S.mitis_NS51_ABS18283.1 PEANQYKPATPVTNPYAVRNRIGKDGKVLPDVFGNGTHVRVHVKDIQGNPVAGAKVAALV

AbpN_S.mitis_SK137_WP_045597421.1 APENQYKPETPAYNPYHG----------KPVVFGNGSHVRFHVRDLQGASVAGVKVDIMV

AbpN_S.mitis_SK145_WP_045606492.1 PADQQYKPETPAYNPYHG----------KPAVFGNGSHVRFSVKDIQGNPIAGVKVDVMV

:**** **. *** * *****:***. *:*:** :**.** :*

AbpC_S.mitis_NS51_ABS18283.1 YATVEDFDNYKKPALIEAVTDAAGDVEFPLANGGYVVYRIDEAPKGYFVPAKLTVGTL--

AbpN_S.mitis_SK137_WP_045597421.1 WANKADYMNYAKASVVSAESNAAGDVELSVPHGGLVQYRVANVPKGYVVKGMESTNGLYT

AbpN_S.mitis_SK145_WP_045606492.1 WANEADYNNYAKASVVSAESNAAGDVELAVPHKGLVQYRVAYVPKGYFLHKVNRTVHNGK

:*. *: ** * :::.* ::******: : . * * **: .****.: .

AbpC_S.mitis_NS51_ABS18283.1 --------------LDAEEV--------VREGSDMFV--TGELVIEKTDTYNVAKEEWVQ

AbpN_S.mitis_SK137_WP_045597421.1 L-NKTSNGAEIGSIAEGTFVYSGSADLDKDTHADMFTNASVDLVLEKTDSYNVVKDQWVQ

AbpN_S.mitis_SK145_WP_045606492.1 ETGNEGQFDIQGNLYDGTFVYSGSADLDKDGKADMFTNASVDLVLEKTDSYNVVKDQWVQ

:. * :***. : :**:****:***.*::***

AbpC_S.mitis_NS51_ABS18283.1 EETGWKYYASNKAVTGWKQVDGKWFFFNAEGVMQKWWVKDGNTWYYLNGNGEMQTGWLQD

AbpN_S.mitis_SK137_WP_045597421.1 EEDGWKYYASNKAVSGWKQVDGKWFFFNAEGVMQKWWVKDGNTWYYLNGNGEMQTGWLQD

AbpN_S.mitis_SK145_WP_045606492.1 EEDGWKYYASNKAVSGWKQVDGKWFFFNAEGVMQKWWVKDGNTWYYLNGNGEMQTGWLQD

** ***********:*********************************************

AbpC_S.mitis_NS51_ABS18283.1 GGKWYYLETSGAMKASQWFEVGGKWYYVDGFRCPCS-----------------

AbpN_S.mitis_SK137_WP_045597421.1 GGKWYYLENSGAMKASQWFTVGGKWYYVDGSGALAVNTTVGGYTVNGNGEWVK

AbpN_S.mitis_SK145_WP_045606492.1 GGKWYYLETSGAMKASQWFEVGGKWYYVDGSGALAVNTTVGGYTVNGNGEWVK

********.********** ********** . .

Blue, signal sequence: red, N-terminal sequence
