# Supplementary figures and images for: Comparative genomics and evolution of the amylase-binding proteins of oral streptococci
Source: BMC Microbiol. 2017 Apr 20;17:94. doi: 10.1186/s12866-017-1005-7 (PMC5399409; doi:10.1186/s12866-017-1005-7)

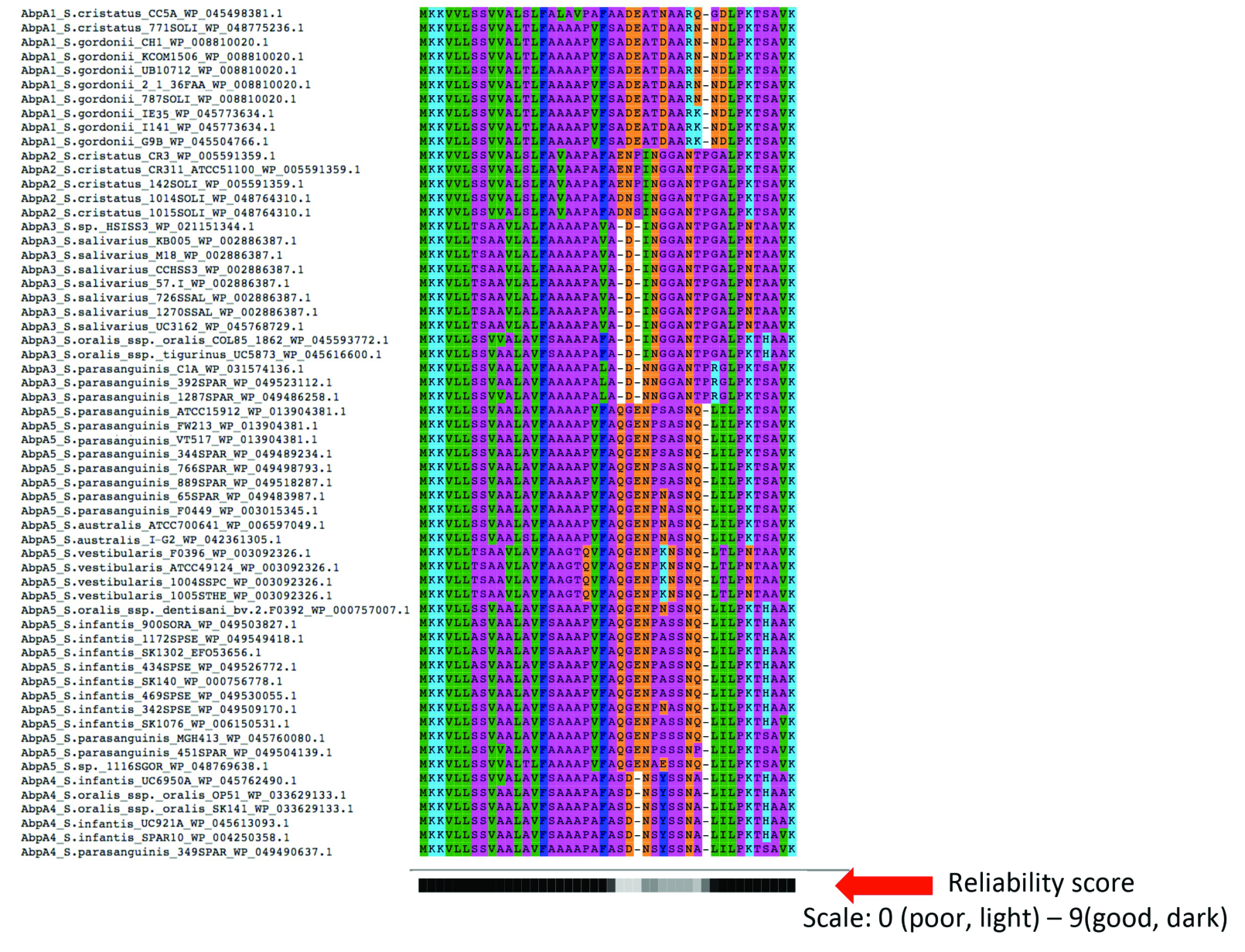

Supplement: Supplementary file 7 — PRANK reliability annotation of the alignment of functional regions (signal sequence and N-terminal sequence) from AbpA-like proteins. (TIFF 11836 kb) [file 12866_2017_1005_MOESM7_ESM.tif]

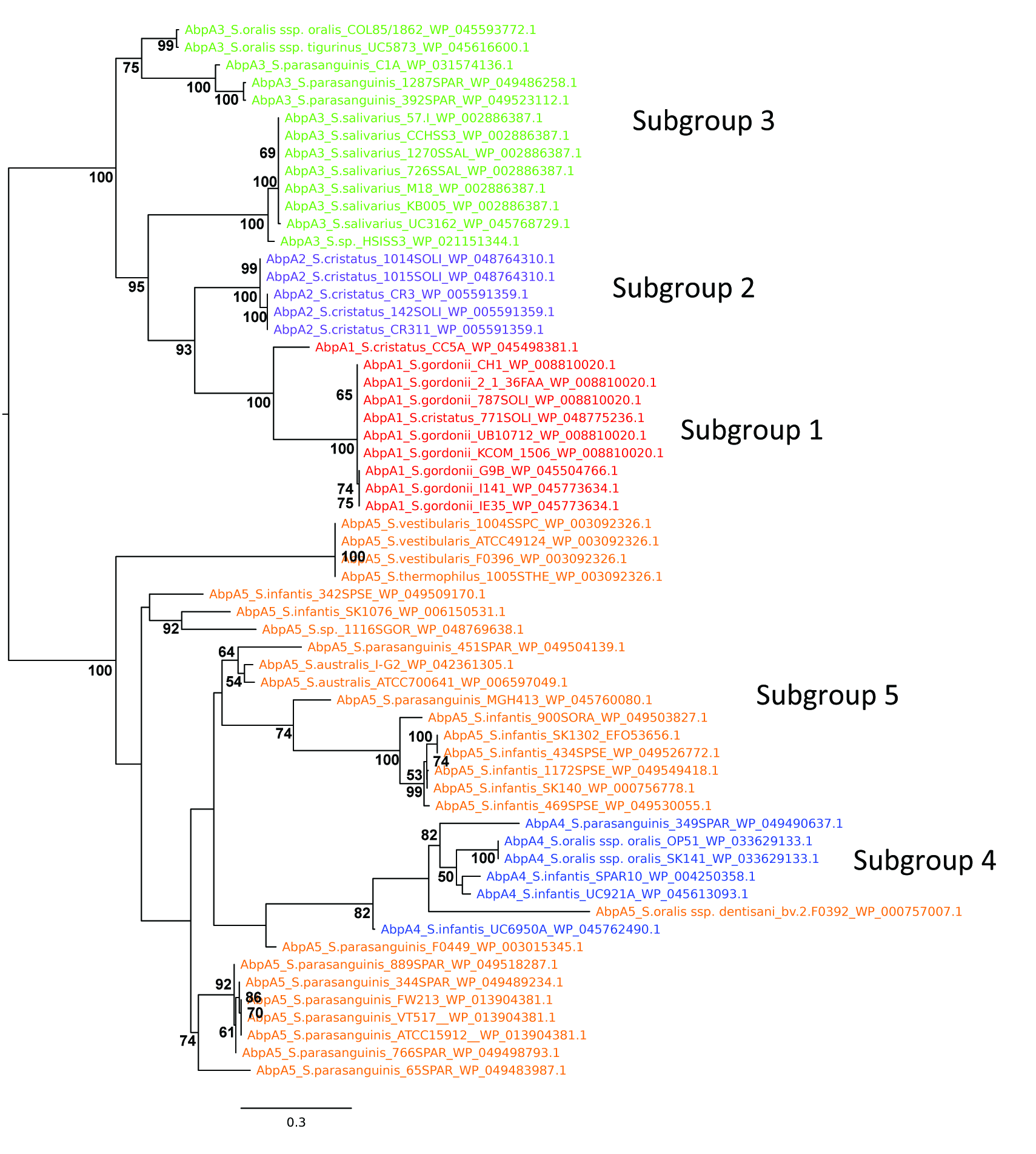

Supplement: Supplementary file 8 — PRANK tree of AbpA subgroups using the entire gene sequence. (TIFF 14602 kb) [file 12866_2017_1005_MOESM8_ESM.tif]

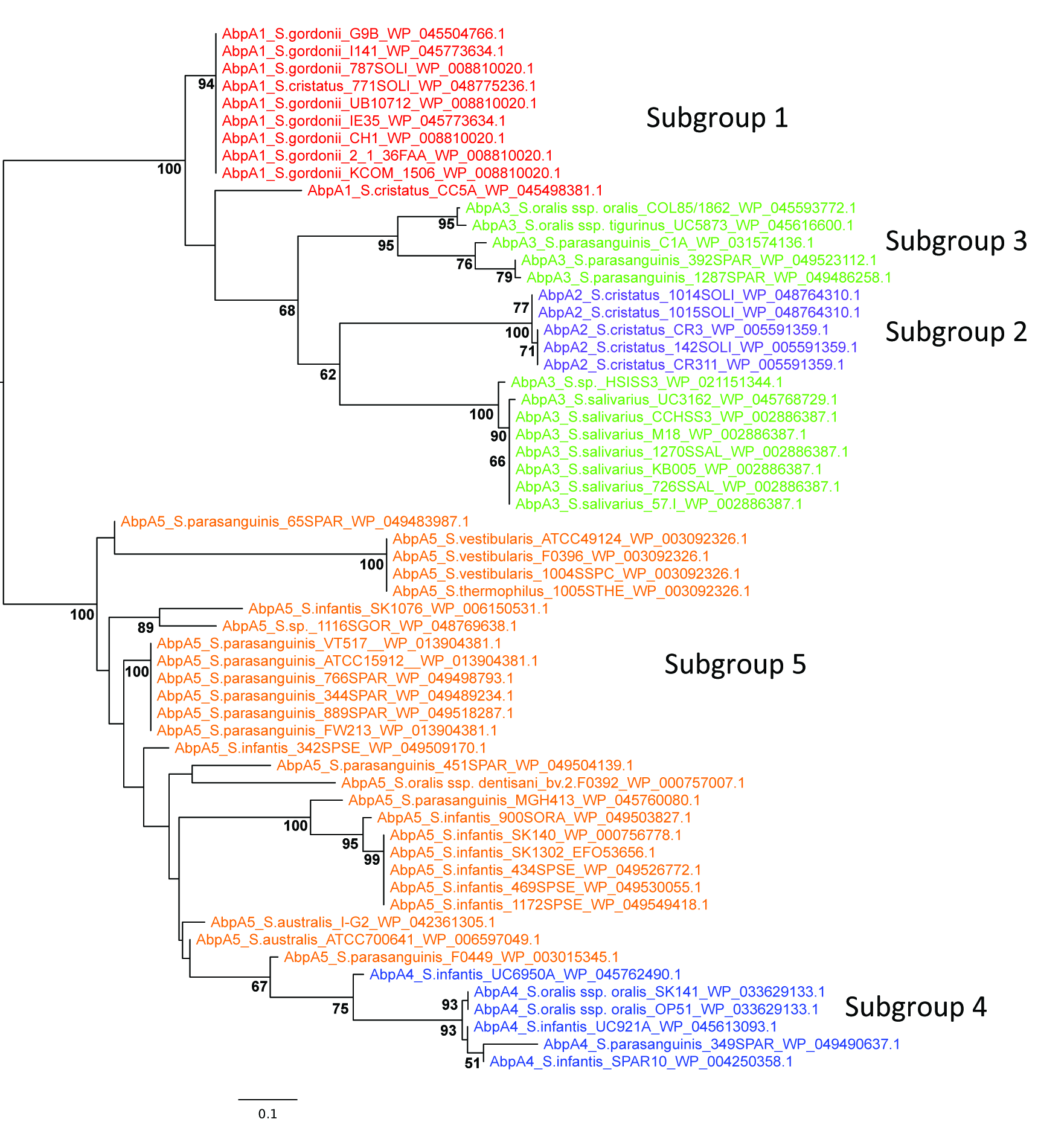

Supplement: Supplementary file 9 — PRANK tree of AbpA subgroups using the entire gene sequence, reliability score 7. (TIFF 14758 kb) [file 12866_2017_1005_MOESM9_ESM.tif]
