# Supplementary material for: Comparative genomics and evolution of the amylase-binding proteins of oral streptococci
Source: BMC Microbiol. 2017 Apr 20;17:94. doi: 10.1186/s12866-017-1005-7 (PMC5399409; doi:10.1186/s12866-017-1005-7)
Supplement: Supplementary file 10 — Degenerate PCR primers for genes encoding AbpB-like proteins. (DOCX 73 kb) [file 12866_2017_1005_MOESM10_ESM.docx]

**Table S5** Degenerate PCR primers for genes encoding AbpB-like proteins

| Primers | | Forward and reverse primers  (5’-3’) | | Template Size  (base pairs) | | Annealing Temperature (°C) | |
| --- | --- | --- | --- | --- | --- | --- | --- |
| Primer set 1 | | F: CRSYABYTGRGKCARYMCTA  *(CRSCASTTGRGKCARTCCTA)  R: TTACTACCATCMCCACGAGC | | 850 | | 52 | |
| Primer set 2 | | F: TCGTGGMGATGGTAGTAACG  R: RTCTTCTGGSACYTTHACMG | | 328 | | 50 | |
| Primer set 3 | | F: CKGTDAARGTSCCAGAAGAY  R: CCRAGCCATAMMKTACCACC | | 544 | | 50 | |
| Primer set 4 | | F: GGKRSWAAYGATRCTGTTCG  R: TKYTGTAGCTGCATARCCRA | | 732 | | 51 | |
| Primer set 5 | | F: TATCGYGCWTCATWRCCAGA  *(TBAAYAAARACHCARCGMGTH)  R: TCAATAARACMCARCGMGTC | | 712 | | 52 | |

R=A+G, Y=C+T, M=A+C, K=T+G, S=C+G, W=A+T, H=A+T+C, B=T+C+G, D=A+T+G, V=A+C+G

* Alternative primers used as needed for different strains.
